# Photopolymerizable Imidazolium Methacrylate Networks for Controlled Dexamethasone Release: Potential Application in Uveitis Therapy

**DOI:** 10.1021/acsomega.5c12938

**Published:** 2026-04-27

**Authors:** Maria J. S. Lima, Dayane K. D. N. Santos, Janaína V. dos Anjos, Severino Alves Junior

**Affiliations:** Departamento de Química Fundamental, 28116 Universidade Federal de Pernambuco, 50740-560 Recife, PE, Brazil

## Abstract

This study focuses on the development of photopolymerizable systems using imidazolium methacrylate, a liquid salt made from imidazole and methacrylic acid, cross-linked with either cyclohexanedimethanol dimethacrylate (CDMM) or ethylene glycol dimethacrylate (EGDMA). These systems are specifically designed for the controlled release of dexamethasone in ocular applications. The swelling behavior, mechanical characteristics, drug release kinetics, and biocompatibility of the resulting materials on different cell lines were evaluated. The dexamethasone release process was governed by a combination of diffusion and polymer matrix relaxation, influenced by drug–polymer interactions and temperature. Implants containing higher dexamethasone concentrations exhibited a release mechanism predominantly controlled by polymer relaxation, associated with chain reorganization. The results revealed that the release profiles varied with the cross-linking agent used and showed significant temperature dependence. At average body temperature, increased matrix swelling and faster release rates were observed. It was observed that higher drug concentrations led to greater porosity, thereby promoting faster release. Conversely, cross-linking agents with lower steric hindrance produced denser networks, resulting in reduced release rates. All tested formulations showed good cytocompatibility. These findings highlight the potential of imidazolium methacrylate–based photopolymerizable systems for applications in personalized medical devices, particularly for controlled and/or localized corticosteroid delivery.

## Graphical abstract

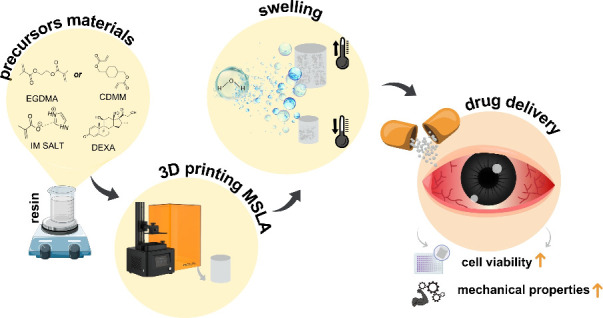

## Introduction

Uveitis is the noun given to a group of inflammatory disorders that affect the uveal tract and may also involve adjacent ocular tissues. If not properly treated, it can lead to severe complications, including vision loss.^1^ The disease is classified according to the primary site of inflammation: in intermediate uveitis, the vitreous body is mainly affected, while in posterior uveitis, the inflammation involves the retina or choroid. Moreover, uveitis can be either infectious or noninfectious, depending on its etiology.^2,3^

The main goals of uveitis treatment are to suppress intraocular inflammation, relieve discomfort, and prevent vision-threatening complications. Corticosteroids, such as dexamethasone, are widely used in uveitis due to their potent anti-inflammatory effects.^4^ However, when administered systemically, corticosteroid therapy often requires high doses over prolonged periods to achieve therapeutic efficacy, leading to significant adverse effects, including weight gain, hypertension, hyperglycemia, gastritis, opportunistic infections, and even psychosis. These side effects can compromise patient adherence and quality of life, highlighting the need for alternative strategies that minimize systemic exposure.^5^

To address these challenges, increasing attention has been directed toward controlled drug delivery systems capable of administering drugs directly to the target site. Such approaches aim to enhance therapeutic efficacy while reducing systemic toxicity. Intraocular and periocular injections represent viable alternatives, allowing high local drug concentrations with fewer systemic effects. Nonetheless, they are invasive, may cause complications, and often require repeated administration.^2,3^

In response, sustained-release drug delivery devices have been developed to provide long-term therapeutic effects while minimizing the risks associated with repeated injections. Recent advances in manufacturing technologies, particularly three-dimensional (3D) printing, have opened new possibilities for designing such devices. 3D printing offers high flexibility in implant design, enabling the fabrication of complex, patient-specific structures. This technology has already found numerous applications in medicine, including ophthalmology. It is expected to play a transformative role in the pharmaceutical field^6–10^ due to its potential for producing personalized medicines, tailored drug-release systems, and customized medical devices.

Among the various 3D printing methods, vat photopolymerization stands out for its high precision and surface quality. It creates solid objects through layer-by-layer photopolymerization of liquid resins under light irradiation. This technique operates at room temperature, avoiding thermal degradation of thermolabile drugs and ensuring structural integrity during printing.^7,11,12^

(Meth)­acrylate-based polymers are the most widely used materials for manufacturing parts via vat polymerization.^13^ Despite the high polymerization rates and good mechanical properties of poly­(meth)­acrylates, their organoleptic properties and lack of biocompatibility can hinder their use in implants.^14^ To overcome these issues, our research group has been working with systems composed of imidazolium methacrylate, an odorless methacrylate ionic liquid that polymerizes rapidly, remains water-soluble even after polymerization, and is cytocompatible.^15,16^ As cross-linkers, several cytocompatible organic methacrylates can be used in different ratios relative to the main polymer, such as cyclohexanedimethanol dimethacrylate,^17^ ethylene glycol dimethacrylate,^18^ and resorcinol dimethacrylate,^15^ among others.

Therefore, this work investigates the potential of 3D printing for the development of controlled-release dexamethasone implants using cross-linked imidazolium polymethacrylate as the polymeric matrix for the treatment of uveitis. By combining additive manufacturing with pharmaceutical formulation principles, the study aims to design and evaluate 3D-printed ocular devices that provide sustained drug release, reduce complications, and enhance therapeutic outcomes.

## Experimental Section

### General

The following chemical compounds were obtained from commercial sources and used in this study: methacrylic acid, imidazole, 2,4,6-trimethylbenzene diphenylphosphine oxide (TPO), sodium chloride, potassium chloride, potassium hydroxide, dibasic sodium phosphate, monobasic potassium phosphate, cyclohexanediol (a mixture of cis and trans isomers), methacrylic anhydride (MA), pyridine, 4-dimethylaminopyridine (DMAP), and silica gel 60 for column chromatography (Sigma-Aldrich, Darmstadt, Germany). Hexane, ethyl acetate, hydrochloric acid, anhydrous sodium sulfate, and sodium bicarbonate were purchased from Labsynth (Diadema, Brazil). Dexamethasone (C_22_H_29_FO_5_, 392.46 g·mol^–1^) tablets (4 mg, commercial name *Dexametasona*) were obtained from a local pharmacy. All reagents were used without further purification, except for pyridine, which was dried over potassium hydroxide and distilled prior to use. The synthesis of the monomers was monitored by thin-layer chromatography (TLC) using GF 254 plates (Sigma-Aldrich, Darmstadt, Germany). Melting points were determined using an Electrothermal Mel-Temp apparatus. ^1^H and ^13^C NMR spectra were recorded on a Varian UNMRS 400 MHz spectrometer using CDCl_3_ (Sigma-Aldrich, Darmstadt, Germany) as solvent. The statistical analysis was performed using GraphPad Prism 9 software, and the graphs were plotted using either Origin 2018 or GraphPad Prism 9 software.

### Synthesis of Monomers

#### Synthesis of Imidazolium Salts

The synthesis of imidazolium salts was carried out as previously reported by de Albuquerque et al.^19^ Imidazole (6.8 g, 0.1 mol) was added to methacrylic acid (8.2 mL, 8.6 g, 0.1 mol) under cooling in an ice bath, affording imidazolium methacrylate. After adding the reagent, the mixture was stirred in the dark for 30 min. The resulting imidazolium salt, a liquid at room temperature (∼25 °C), was used directly for resin preparation without purification.

#### Synthesis of Cyclohexanedimethanol Dimethacrylate (CDMM)

The synthesis of CDMM followed the procedure described by dos Santos et al.,^17^ and its structure was consistent with the expected methacrylate (see S3). Cyclohexanedimethanol (14.4 g; 0.1 mol), 30 mL of pyridine, 40 mL of methacrylic anhydride (MA), and 125 mg (0.001 mol) of DMAP were added to a round-bottom flask. The mixture was stirred for 24 h at room temperature. After this period, the solution was washed with distilled water and then acidified with hydrochloric acid to neutralize the pyridine. To extract the organic product, 40 mL of ethyl acetate was added in two portions (2 × 40 mL). The organic phases were separated and washed with a saturated sodium bicarbonate solution, then dried over anhydrous sodium sulfate. The solvent was removed using a rotary evaporator. The resulting residue was purified by column chromatography, using silica gel 60 (70–230 mesh) as the stationary phase and a hexane: ethyl acetate mixture (7:3, v/v) as the mobile phase. This process yielded 19.1 g of CDMM as a colorless solid, with a 68% yield (Scheme 1).

**Scheme 1:**
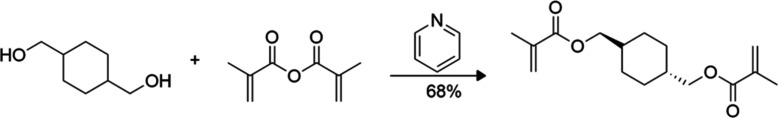
Synthesis of CDMM

#### Synthesis of Ethylene Glycol Dimethacrylate (EGDMA)

The synthesis of EGDMA was performed according to the procedure described by Chan et al.,^20^ and its structure matched the expected methacrylate (see S3). For the synthesis of EGDMA, 5.59 mL (0.1 mol) of ethylene glycol was added to a 250 mL round-bottom flask containing 20 mL of pyridine, under magnetic stirring and cooling in an ice bath. After a few minutes of stirring, 37.93 mL of methacrylic anhydride (0.25 mol) and 0.0122 g of DMAP (0.1 mol % %) were added. The reaction was stirred for 48 h. After this period, the mixture was washed with distilled water and then acidified with hydrochloric acid to neutralize the pyridine. For extraction, 30 mL of ethyl acetate was added twice. The organic phase was separated and washed with a saturated solution of sodium bicarbonate. The organic phase was then dried over anhydrous sodium sulfate, and the solvent was removed using a rotary evaporator. Finally, the residue was left under vacuum until a constant weight was achieved, resulting in 17 g of ethylene glycol dimethacrylate as a colorless oil, with a yield of 73% (Scheme 2).

**Scheme 2:**
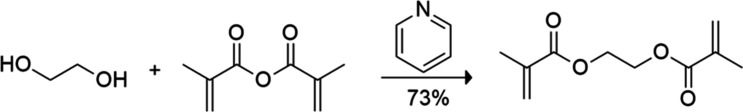
Synthesis of EGDMA

### Extraction of Dexamethasone from Tablets

For the extraction of dexamethasone, tablets were crushed into fine, white powder. For every 100 crushed tablets, 400 mL of ethanol was added to the powder, and the mixture was stirred for 2 h at room temperature. The material was then filtered and washed three times with ethanol to remove the excipients, namely starch, mannitol, magnesium stearate, and povidone. The solvent was subsequently evaporated using a rotary evaporator, and the resulting powder was recrystallized from ethanol and water, yielding a recovery of 96%. The obtained dexamethasone was analyzed by ^1^H NMR using DMSO_d6_ (deuterated dimethyl sulfoxide), and its structure was confirmed in accordance with the expected results (data available in the Supporting Material).

### Resin Preparation

Five resin formulations were developed, varying both the cross-linking agent and the amount of incorporated drug. The preparation protocol followed these steps: initially, 2,4,6-trimethylbenzoyl-diphenylphosphine (TPO; 2.72 × 10^–4^ mol; 0.01 eqiuv; 1 mol %; 0.095 g) was weighed as the photoinitiator and mixed with imidazolium methacrylate (2.72 × 10^–2^ mol; 1 equiv; 4.2 g) in a 20 mL beaker. Next, the cross-linking agent was added. For the formulations containing EGDMA (2.72 × 10^–4^ mol; 0.01 equiv; 1 mol %; 0.054 g), the cross-linking agent was incorporated into the mixture and combined with 40 mg of dexamethasone. The procedure was similar for the formulations containing CDMM (2.72 × 10^–4^ mol; 0.01 equiv; 1 mol %; 0.068 g). In this case, the amount of drug varied: 40, 80, 100, or 220 mg. After adding the components, the mixtures were stirred at room temperature and kept in a dark condition to prevent premature polymerization. To ensure homogeneity, an ultrasonic bath (Branson B2510E-DTH operating at 40 kHz) was used. The resins obtained upon incorporation of 40 mg of drug formed implants containing 0.126 mg of dexamethasone. For formulations containing 80 mg of drug, each implant contained approximately 0.252 mg of dexamethasone; for formulations containing 100 mg, 0.315 mg per implant; and for formulations containing 220 mg, 0.690 mg per implant. This was the maximum concentration that could be dissolved in the amount of resin used in each vat polymerization (2 mL). Different concentrations below this limit were tested to evaluate whether variations in concentration affect the release kinetics. The amount of dexamethasone in each implant was determined based on the volume of the implants (0.012 mL). The formulations and the names associated with each resin are detailed in Table 1.

**Table 1:** Composition and Designation of Resin Formulations

**resin designation**	**dexamethasone concentration**(mg/mL)	**cross-linker**	**drug amount per implant** (mg)
Res C		CDMM	
Res E		EGDMA	
Res 1	20	CDMM	0.126
Res 2	40	CDMM	0.252
Res 3	50	CDMM	0.315
Res 4	110	CDMM	0.690
Res 5	20	EGDMA	0.126

### 3D Printing

Implants with dimensions of 2.5 × 2.5 mm and a layer thickness of 0.05 mm were printed using an AnyCubic Photon MSLA 3D printer with 4K resolution (China), enhanced by a custom-modified prototype vat designed to minimize resin volume (2 mL). The models of the modified vat and platform were developed by us, and the corresponding files are available at cults3d.com.^21^ Additionally, for the mechanical tests, samples measuring 30 × 4 × 2 mm (size 1BB) were printed, as well as samples with dimensions of 6.0 × 6.0 mm and a layer thickness of 0.05 mm for Shore hardness testing. All objects were printed using the same printing parameters: 15 s of exposure per standard layer and 45 s for the first five base layers. Meshmixer software was used for object projection, and the file was imported into the printer’s software in.pwma file format. After each printing cycle, the implants were washed with isopropyl alcohol for 1 min and then cured using a AnyCubic Wash and Cure 2.0 (China) for 1 min. For the subsequent mechanical tensile tests, the dimensions of each tensile bar, including total length, total width, and thickness, were measured using a digital caliper.

### Printed Objects’ Characterization

The dimensions of the objects were determined using a digital caliper (KingTools, Brazil). Shore D hardness was measured using a portable digital durometer (Romacci, Brazil) on 6.0 × 6.0 mm cylinders, in accordance with ASTM D2240 standards. Fourier Transform Infrared Spectroscopy (FTIR) was performed using a diamond crystal (Bruker Alpha-II, USA) in ATR mode. Mechanical testing was conducted using specimens for tensile and stress testing, in accordance with ISO 527 standards. Measurements were made with the TRD 22 probe and the Emic equipment (Instron, Brazil). Thermogravimetric curves (TG) were obtained using a TGA-50H (Shimadzu, Japan) under a nitrogen atmosphere with a heating rate of 10 °C/min. Macroscopic images were captured using a USB microscope with an optical zoom of up to 1600x (Universal Serial Bus), a Play 16 (PlayShop, Brazil), or a Moticam SMZ-161 optical microscope (Motic, China). For microscopic images, a scanning electron microscope (SEM), Tescan Mira 3 (Tescan, Czech Republic), was used, operating with an acceleration voltage ranging from 5 to 10 kV to assess the morphology and surface of the implants.

### Swelling Tests

In this test, the specimens (2.5 × 2.5 mm cylinders) were immersed in a container containing approximately 10 mL of phosphate-buffered saline (PBS). This test aimed to identify prototypes that exhibited mass changes over 5 days. The mass increase was calculated based on the cylinder masses at the time of measurement compared to the measurement at day 0, using the following formula:

$$S\;(\%)=\frac{M_{f}-M_{i}}{M_{i}}\times 100\%$$ 1

where *S* (%) is the swelling rate, *M*_f_ is the mass of the sample after the immersion period, and *M*_i_ is the initial dry mass of the sample. The swelling studies were conducted at room temperature (25 °C) and at 35.7 °C, the average human body temperature. Swelling tests were performed at pH 7.4 and pH 8.0. All swelling experiments were performed in triplicate (*n* = 3) to ensure reproducibility.

### Preparation of Dexamethasone Working Solutions for the Calibration Curve

A stock solution of dexamethasone (0.4 mg/mL) was used. Working solutions were prepared by serial dilutions of the stock solution in phosphate-buffered saline (PBS). Each solution was transferred to a 10 mL volumetric flask and diluted with PBS. Seven different concentrations of dexamethasone (0.015, 0.0075, 0.0035, 0.0015, 0.00095, 0.00047, and 0.00024 mg/mL) diluted in PBS were prepared to estimate absorbance and construct a calibration curve. This study used an Agilent Cary 60 UV–vis spectrophotometer (Agilent, USA). After serial dilution with different buffers, solutions containing various concentrations of the compound dexamethasone were scanned from 200 to 400 nm to determine the maximum wavelength (λ_max_). The solutions showed maximum absorption at 240 nm.

### Dexamethasone Release Tests

All materials used in the procedure, such as glassware, resins, and Milli-Q water, were sterilized in an autoclave. The drug-loaded cylindrical printed resins were placed in flat-bottomed vials containing 10 mL of phosphate-buffered saline (PBS), and the release test was initiated. The vials were placed on a Kline shaker (SPLabor, Brazil) at 200 rpm. The test was conducted at ambient temperature (25 °C) and at 35.7 °C (the average human body temperature). The system was monitored until complete drug release occurred. At predefined time intervals, 120 μL of the sample was withdrawn and diluted in 2 mL of Milli-Q water. The solution was then analyzed using a spectrophotometer to determine absorbance and calculate the concentration of the released drug over time. The cumulative drug release percentage was determined and plotted as a function of time. Each drug release test was performed in triplicate.

For a more detailed analysis of the release mechanism, the experimental data were fitted to the Korsmeyer–Peppas model. Korsmeyer et al.^22^ derived a simple equation that describes drug release from a polymeric system, as shown below:

$$\mathrm{Korsmeyer}-\mathrm{Peppas}\;\mathrm{mod}\mathrm{el}:\frac{Q_{t}}{Q_{\infty }}=k_{Κ}\times t^{n}$$ 2

where *Q*_*t*_ is the amount of drug released at time *t*, $\frac{Q_{t}}{Q_{\infty }}$ represents the fraction of drug released at time *t*, *k*_Κ_ is the release rate constant for the Korsmeyer–Peppas model, and *n* is the release exponent that indicates the release mechanism.

### Implants Cytocompatibility Test

#### Cytotoxicity Assay on VERO Cells

Cytotoxicity was evaluated using the MTT assay [3-(4,5-dimethylthiazol-2-yl)-2,5-diphenyl-2H-tetrazolium bromide] on the VERO cell line (ATCC CCL-81). Cells (1 × 10^6^ cells/mL) were cultured in RPMI 1640 medium supplemented with 10% fetal bovine serum and 1% antibiotic solution and seeded into 24-well plates. After 24 h of incubation at 37 °C in a humidified 5% CO_2_ atmosphere, a cylindrical resin specimen (2.5 × 2.5 mm) containing dexamethasone was added to each well and incubated for an additional 24 h. Following incubation, 10 μL of MTT solution was added to each well, and the plates were incubated for 4 h. The medium was then removed, and the formazan crystals formed were dissolved in 100 μL of DMSO. Absorbance was measured at 595 nm using a microplate reader (Thermo Scientific Multiskan SkyHigh Spectrophotometer). Cells cultured in medium only served as the negative control. All experiments were performed in triplicate.

#### Cytotoxicity Assay on SIRC Cells

Cytotoxicity was evaluated using the MTT assay [3-(4,5-dimethylthiazol-2-yl)-2,5-diphenyl-2H-tetrazolium bromide] on SIRC cells (Statens Serum Institut Rabbit Cornea). Cells (1 × 10^5^ cells/mL) were cultured in Dulbecco’s Modified Eagle Medium (DMEM) supplemented with 10% fetal bovine serum and 1% antibiotic solution and seeded into 24-well plates. After incubation for 24 h at 37 °C in a humidified 5% CO_2_ atmosphere, a cylindrical resin specimen (2.5 × 2.5 mm) containing dexamethasone was added to each well and incubated for an additional 24 h. Following incubation, 50 μL of MTT solution was added to each well, and the plates were incubated for 4 h. The medium was then removed, and the resulting formazan crystals were dissolved in 500 μL of DMSO. Absorbance was measured at 595 nm using a microplate reader (Thermo Scientific Multiskan SkyHigh Spectrophotometer). Cells cultured in medium only served as the negative control. All experiments were performed in triplicate.

## Immediate Release Assay of Dexamethasone from the Printed Matrix

A unit of the printed specimen containing the drug, measuring 2.5 × 2.5 mm, was macerated in a mortar and pestle until completely pulverized. Then, 10 mL of PBS was added to this powder and subjected to ultrasonic treatment (Branson B2510E-DTH ultrasonic bath operating at 40 kHz, Branson Ultrasonics, USA) for 30 min to extract the dexamethasone. After this period, 60 μL of this PBS solution was diluted with more PBS to form a 2 mL solution. This diluted solution was homogenized and analyzed using an Agilent Cary 60 UV–vis spectrophotometer (Agilent, USA) at 242 nm. Drug concentration was measured using a calibration curve of dexamethasone. This assay was carried out in triplicate.

## Results and Discussion

The fabrication of the implants for the controlled release of dexamethasone was carried out in two steps: (1) preparation of the main imidazole methacrylate network cross-linked with ethylene glycol dimethacrylate (EGDMA) or cyclohexanedimethanol dimethacrylate (CDMM) and incorporated with varying amounts of the drug, followed by (2) controlled drug release testing associated with cytotoxicity and mechanical assays.

These two monomers were selected as cross-linkers because they contain two methacryloyl groups, are liquids with suitable viscosity, and produce biocompatible polymers. In addition to EGDMA, which is already widely used in dental materials, for example, CDMM was also selected. Because it is cyclic and more rigid, CDMM may impart different properties to the final polymeric matrix.

In previous studies, our group developed and printed resins containing only polymerizable imidazolium salts, which exhibited rapid dissolution in water after just a few minutes of immersion.^19^ This rapid dissolution is unsuitable for controlled drug delivery systems, as it would lead to immediate drug release. To address this issue, we introduced cross-linking agents such as EGDMA and CDMM. The addition of these agents resulted in a reduction in the solubility of the printed polymer matrix, enabling the devices to function as a network for gradual drug release. As bodily fluids penetrate the polymer structure, its layers gradually move apart, releasing the drug in a controlled manner.

The cross-linking agents, containing two methacrylation sites, were evaluated for their effect on modifying the release properties. These agents were employed at a concentration of 1 mol % relative to the imidazolium salt, which is responsible for forming the main polymeric network of the system. It was observed that increasing the cross-linker content led to the formation of rigid, nonhydratable, and nonswellable structures. In contrast, formulations containing less than 1 mol % cross-linker, or without a cross-linker, resulted in materials that completely disintegrated upon immersion in aqueous medium.

We also investigated the effect of increasing dexamethasone concentration in implants developed with CDMM, as implants with EGDMA did not support higher drug concentrations in previous tests. Other important factors assessed included mechanical properties (as determined by tensile tests), swelling behavior, and drug release patterns. All developed devices were printed successfully, resulting in cylindrical specimens with the intended dimensions. After printing, the dimensions of the samples were verified, and their Shore D hardness (Table 2) was measured using a digital bench durometer. Specimens for the tensile tests were 30 × 4 × 2 mm (size 1BB), while specimens for the hardness test measured 6 × 6 mm. All printed objects are shown in Figure 1. In Table 2, formulations Res C and Res E correspond to the implants containing CDMM and EGDMA, respectively, without drug incorporation.

**Table 2:** Shore D Hardness for Printed Objects with Standard Deviation Values (*n* = 3)

**resin designation**	**shore hardness**
Res C	64.8 ± 2.6 D
Res E	54.1 ± 2.6 D
Res 1	66.2 ± 2.2 D
Res 2	67.1 ± 2.2 D
Res 3	67.3 ± 2.1 D
Res 4	118.5 ± 2.2 D
Res 5	67.1 ± 2.1 D

**Figure 1:**
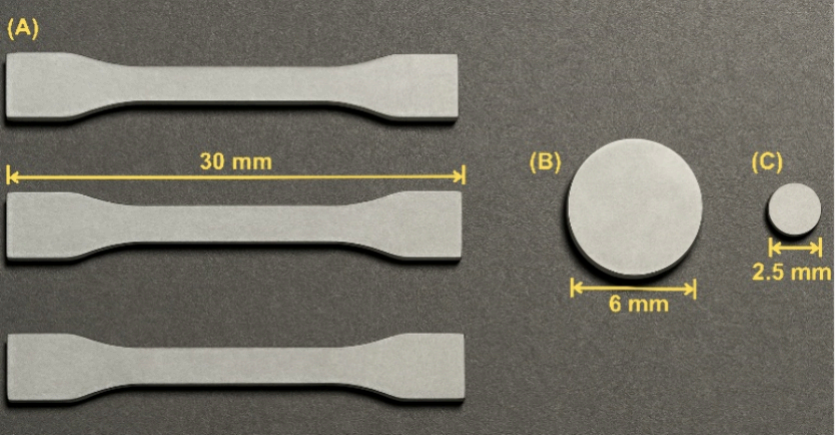
Representation of the printed objects developed in this study. (A) Specimen for mechanical testing, measuring 30.0 × 4.0 × 2.0 mm; (B) Cylinder of 6.0 × 6.0 mm, used in hardness tests; and (C) cylinder of 2.5 × 2.5 mm, employed in drug release, swelling, degradation, and cytotoxicity assays.

Hardness testing is widely used in material characterization, providing a practical and reproducible assessment of mechanical and physical properties. The therapeutic effectiveness and bioavailability of implants are directly related to parameters such as weight uniformity, friability, hardness, and content uniformity, all of which were evaluated after fabrication.^23,24^ Hardness is defined as the resistance of a material to localized surface deformation. According to the Shore hardness scale, materials with values between 61 and 80 D are classified as hard.^25–27^

Based on these parameters, it is possible to design devices with distinct mechanical characteristics tailored to specific requirements. As presented in Table 2, implants without drug loading exhibited Shore D hardness values of 64.8 ± 2.6 for Res C and 54.1 ± 2.6 for Res E. For the specimens containing dexamethasone, an increase in drug concentration was directly correlated with higher Shore hardness in devices produced with CDMM. In this case, the greater the drug concentration, the higher the measured hardness. Resins 1, 2, and 3 exhibited Shore D values above 60. In contrast, resin 4, containing the highest drug concentration, showed hardness exceeding 100 D. The resin containing the cross-linking agent EGDMA (resin 5) also exhibited hardness values above 60 D.

The increase in hardness with higher drug concentrations can be attributed to the layer-by-layer printing process. As the structure is printed as a continuous solid, the greater amount of drug incorporated within the layers influences the mechanical behavior of the device, resulting in a more resistant material.^28^

The mechanical properties of the implants are crucial for evaluating their feasibility and effectiveness in controlled drug release. In addition to the hardness testing, tensile tests were conducted further to characterize the mechanical behavior of the developed implants. The aim was to determine whether increasing the dexamethasone concentration or modifying the cross-linking agent affected these properties. The data obtained are presented in Figure 4, where resins C and E (Res C and Res E) correspond to formulations with CDMM and EGDMA, respectively, without the drug. The stiffness of the implants was assessed using Young’s modulus. Implants with low mechanical strength, exhibiting higher fragility, are at risk of fracturing during the implantation process, making them unsuitable for clinical use.

The mechanical properties of the printed polymers are directly related to the effectiveness of chemical linking at the interfaces between phases. In a binary mixture, as in this study, optimal drug concentration may influence adhesion between the interfaces of the two polymeric phases.^29^ In this context, we evaluated the mechanical properties of five implants containing dexamethasone incorporated into their formulation, as well as two implants fabricated without the drug, for comparison purposes. The primary mechanical property analyzed was stiffness.

According to the results obtained, increasing dexamethasone concentration affected the mechanical behavior of the implants, which is consistent with experimental observations. These observations also indicated that implants produced without the drug exhibited distinct behavior during the printing process, requiring a longer light exposure time for curing standard layers.

Unloaded resins showed excellent mechanical performance, with an elasticity modulus of 813 MPa for those formulated with EGDMA and 1493 MPa for those formulated with CDMM (as shown in Figure 2). These values indicate that the materials are highly rigid and could be suitable for drug delivery applications in the posterior region of the eye. For printed resins containing the drug, the elastic modulus significantly increased (the stiffer the material, the higher the Young’s modulus)^30^ reaching 1764 MPa for CDMM-based resins with higher drug concentrations and 1654 MPa for those containing EGDMA. These values fall within the range reported for filaments composed of ethylcellulose and various release modifiers, such as Soluplus, PEG 6000, Eudragit RL, polyvinyl acetate, and Kollidon, among others.^31^

**Figure 2:**
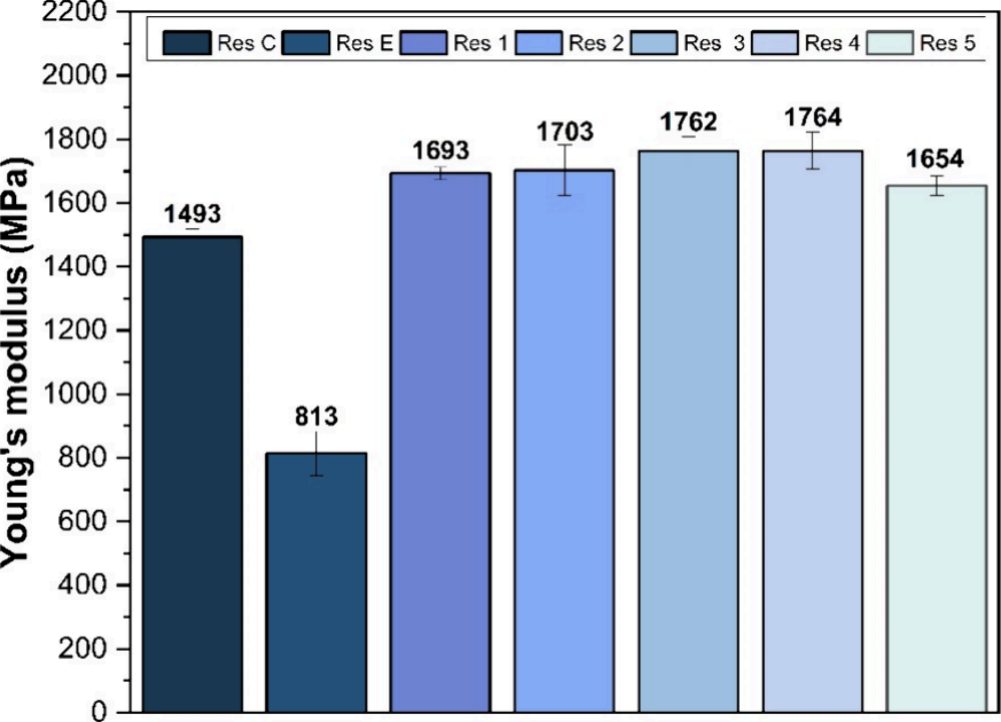
Young’s modulus of the printed specimens with and without dexamethasone (*n* = 3).

Numerous studies have reported that reinforced materials can achieve moduli of 1000 MPa or higher, making them suitable for ocular applications. For instance, PEEK, poly­(ether ether ketone), when reinforced with glass or carbon fibers, can reach these values. Such materials are widely used in medical devices due to their high strength, biocompatibility, and ability to be molded into complex geometries. Although pure PEEK does not reach this range, its composites can be engineered for use in ocular applications, such as structural support implants or intraocular prostheses.^32^

Shape-memory polymers and hydrogel-based composites can also be modified to exhibit enhanced mechanical properties, potentially approaching the rigidity required for applications that demand higher structural strength. Additionally, PLGA, a biodegradable polymer used in the intravitreal implant Ozurdex, which delivers dexamethasone in a controlled manner to the eye, exhibits a Young’s modulus between 1 and 2 GPa, depending on its lactic-to-glycolic acid ratio, degree of crystallinity, and manufacturing conditions. This comparison validates that our implants possess the essential mechanical characteristics required for the proposed ocular application.^33^

The Young’s modulus values did not show significant variations among the drug-loaded resins; however, the incorporation of dexamethasone clearly influences the stiffness of the devices. In general, resins with higher dexamethasone concentrations exhibited higher Young’s modulus values.

In addition to hardness, surface roughness of controlled-release devices can substantially affect drug release kinetics, adhesion to the application site, and interactions with biological tissues. Increased roughness can enlarge the contact area with biological fluids, accelerating the initial release of the active compound.^34^

Moreover, surface roughness can modulate cellular interactions and help regulate the release profile, reducing the initial burst and extending the duration of drug action.^35^ For example, the microstructure of the surface can influence the diffusion of dexamethasone through the device or surrounding tissue, ensuring a more consistent and controlled release over time, which is crucial for maintaining therapeutic efficacy and safety.

Figure 3 illustrates the morphologies of the printed implants, highlighting notable differences between them. Implants containing dexamethasone exhibit evident surface roughness, whereas those without the active compound show smooth, pore-free surfaces. This suggests that the inclusion of dexamethasone has a significant impact on the material’s topography.

**Figure 3:**
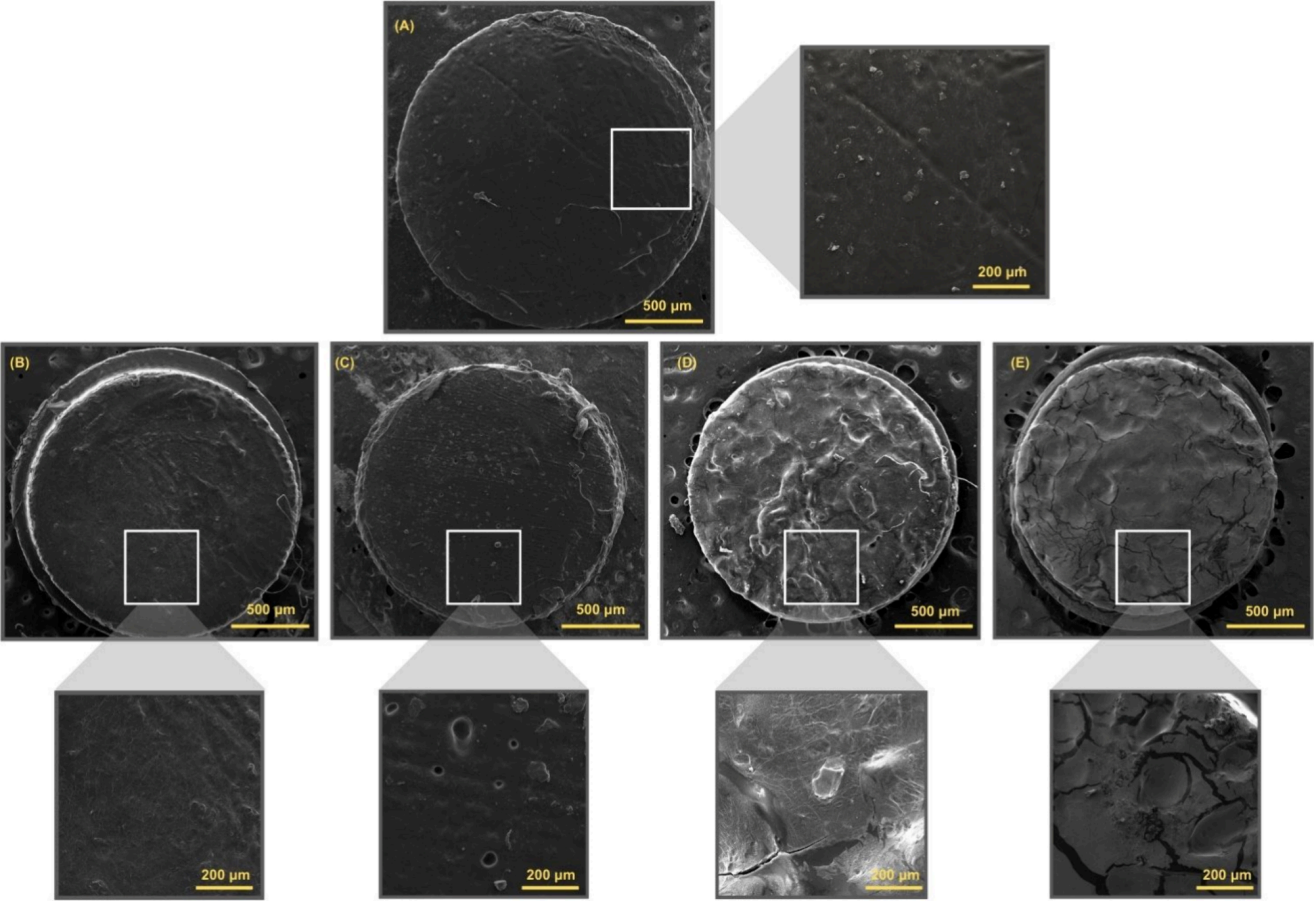
SEM of implants with and without dexamethasone (CDMM): (A) Res C; (B) Res 1; (C) Res 2; (D) Res 3; and (E) Res 4.

As shown in Figures 3 and 4, increasing the concentration of dexamethasone leads to a pronounced increase in surface roughness for implants containing either CDMM or EGDMA, compared to those without the compound. In particular, for CDMM-based implants, higher dexamethasone concentrations clearly contribute to greater surface roughness.

**Figure 4:**
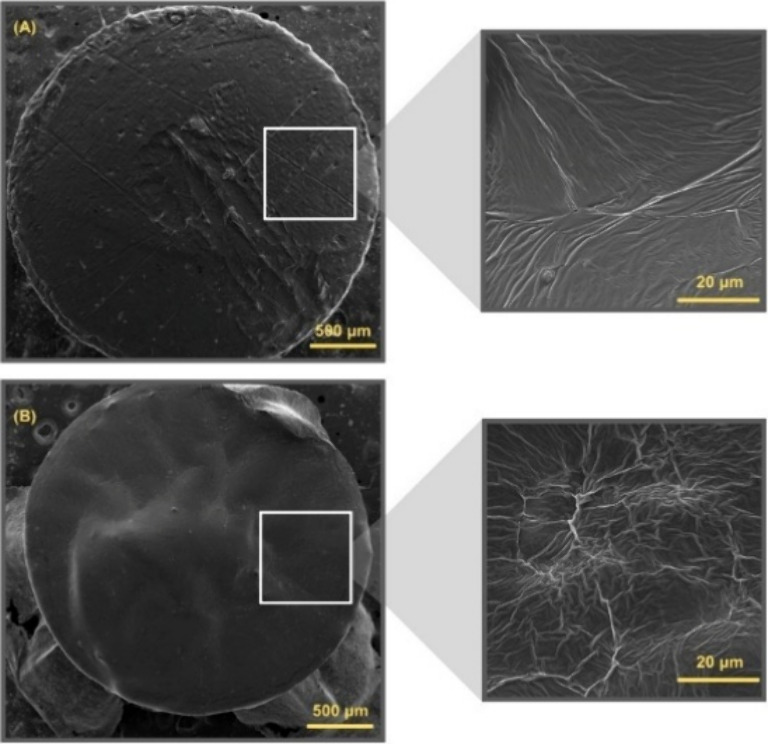
SEM of implants with and without dexamethasone (EGDMA): (A) Res E and (B) Res 5.

These findings suggest that the morphological changes induced by the incorporation of the active compound are directly dependent on its concentration, indicating that this effect may result from interactions between the substance and the polymeric matrix. Further studies are required to investigate factors that may limit the increase in roughness at higher concentrations and to elucidate how this interaction influences the functionality and biocompatibility of the loaded devices.

The FTIR spectra of the dexamethasone-loaded resins exhibit significant differences compared to the precursors, imidazole, methacrylic acid, EGDMA, and CDMM, confirming the successful formation of the material (S6). In the resins containing the cross-linking agent EGDMA or CDMM, an intense band at 1700 cm^–1^ is attributed to the C=O stretching vibration of the ester group, confirming that the methacrylate moiety is bound to the end of the polymer chain through an ester linkage. In addition, a weak signal at 1630 cm^–1^ is assigned to the C=C stretching vibration.^36,37^

It is also important to highlight the changes observed in the spectra of the resins before and after printing, which demonstrate the successful polymerization of the dexamethasone-containing formulations. The signals corresponding to the C=C bonds of the methacrylate group, at 1630 and 815 cm^–1^, exhibited markedly reduced intensities after printing, confirming the formation of the polymeric network as the double bonds turned into single bonds (Figure 5).^38^

**Figure 5:**
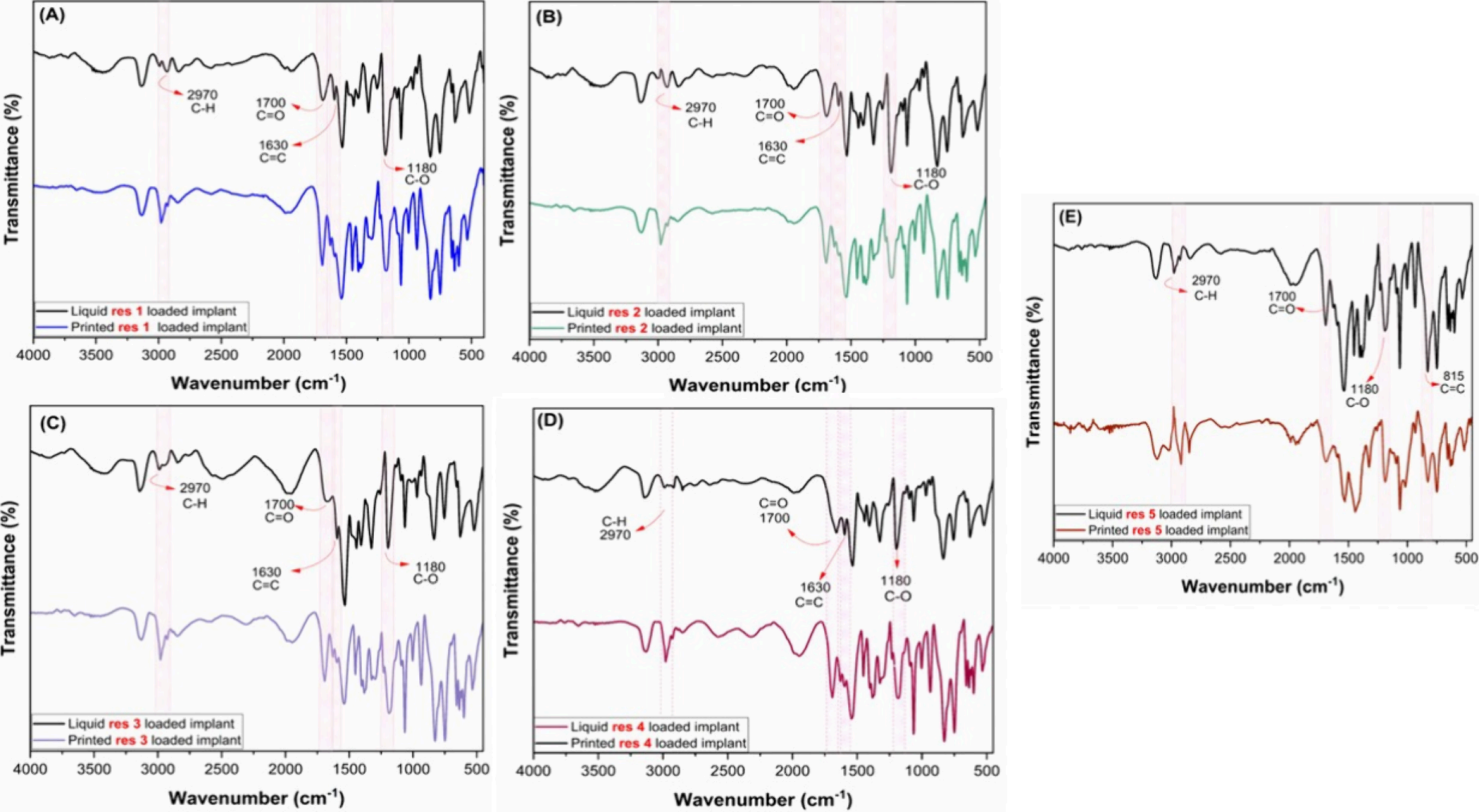
Comparative FTIR spectra of liquid and cured resins: (A) Res 1, (B) Res 2, (C) Res 3, (D) Res 4, and (E) Res 5.

Thermogravimetric analysis (TGA) was employed to investigate the thermal stability of the printed resins (Figure 6). Together with its derivative (DTG), this technique is an essential tool for the thermal characterization of polymeric biomaterials, as it enables the assessment of both thermal resistance and degradation mechanisms.^39,40^ While the TGA monitors mass variation as a function of temperature, the DTG provides the corresponding degradation rates, allowing for the identification of temperatures associated with decomposition events. Each cross-linking agent (EGDMA or CDMM) exhibited a characteristic thermal degradation profile.

**Figure 6:**
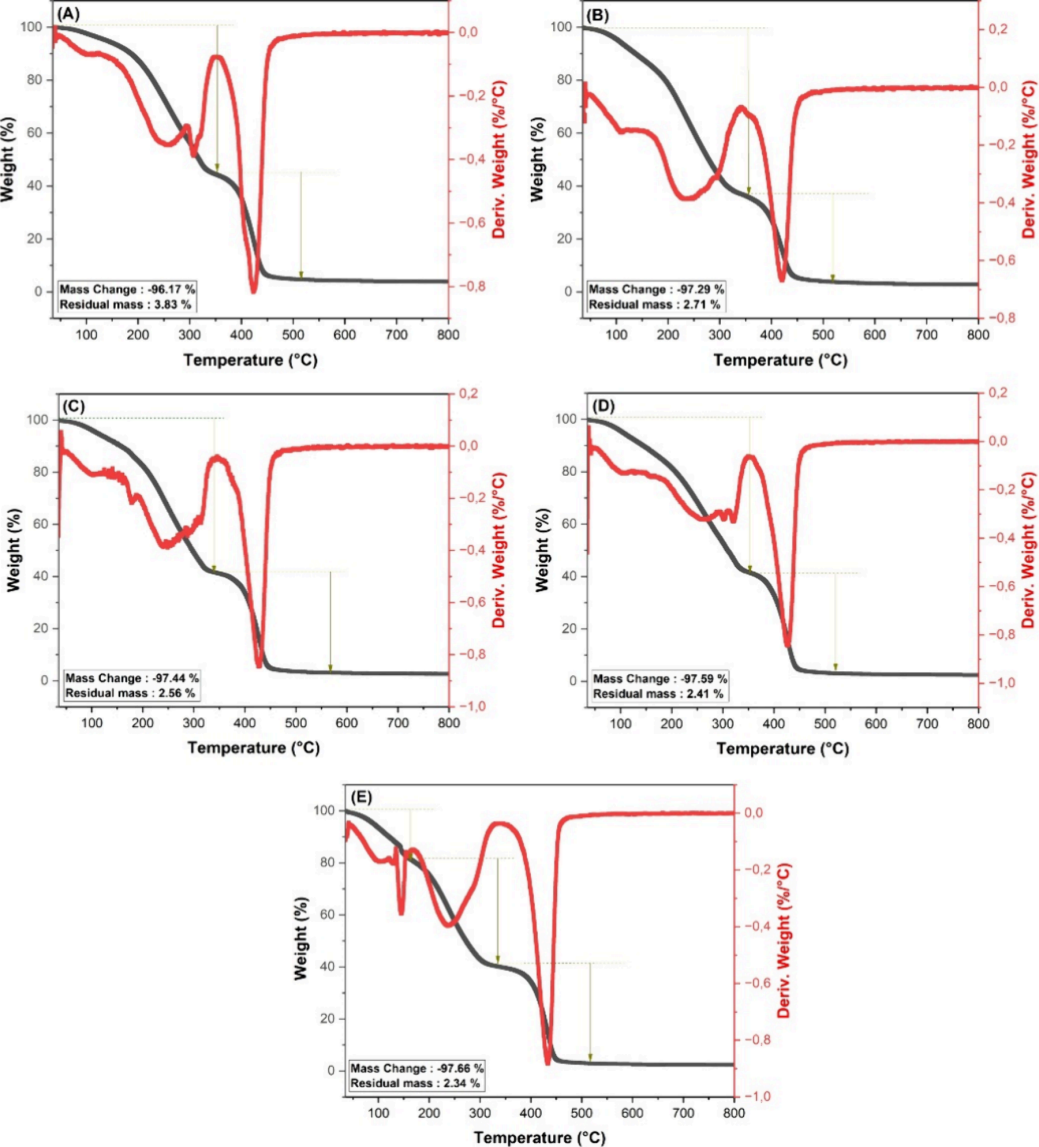
TG and DTG curves of printed resins: (A) Res 1; (B) Res 2; (C) Res 3; (D) Res 4; and (E) Res 5.

Materials formulated with EGDMA typically decompose between 200 and 400 °C due to the cleavage of C–C and C–O bonds in ester groups,^41^ whereas CDMM-based materials tend to exhibit degradation events at higher temperatures, close to 400 °C.^17^

In the specimens developed in this study, two distinct thermal behaviors were observed depending on the cross-linking agent (Table 3). Samples prepared with CDMM (Res 1–4) exhibited two main degradation stages. In contrast, the resin formulated with EGDMA (Res 5) showed three well-defined thermal events, consistent with its more flexible and less rigid polymeric structure.

**Table 3:** Thermal Degradation Stages and Corresponding Mass Losses of Printed Resins

**resin**	**1° stage of thermal degradation (°C)**	**mass loss in the 1° stage** (%)	**2° stage of thermal degradation (°C)**	**mass loss in the 2° stage** (%)	**3° stage of thermal degradation (°C)**	**mass loss in the 3° stage** (%)
Res 1	73.5–344.2	53.84	360.4–453.1	42.33		
Res 2	65.9–327.3	61.45	357.1–452.0	35.84		
Res 3	63.8–336.9	57.70	352.8–457.1	39.74		
Res 4	64.3–347.2	56.60	363.1–456.1	40.99		
Res 5	52.3–163.4	17.71	179.2–334.4	40.60	356.1–458.1	39.35

For the CDMM-based systems, the first degradation event occurred between approximately 64–73 and 327–347 °C, depending on the sample, resulting in a mass loss between 53 and 61% of the initial weight. The second stage, observed between 350 and 457 °C, resulted in an additional mass loss of 35–42%, leaving a final residue of less than 3–4% in all samples.

The first event is likely associated with the decomposition of low-molecular-weight compounds and initial bond scission processes facilitated by the lower thermal resistance of C–C and C–H single bonds,^42^ whereas the second stage corresponds to the degradation of the CDMM polymer backbone. It was also noted that higher dexamethasone content reduced the final residue percentage, suggesting complete thermal decomposition of the drug during heating.^43^

Conversely, the resin prepared with EGDMA (Res 5) exhibited a three-step degradation profile. The first stage occurred between 52.3 and 163.4 °C, with a mass loss of 17.7%, attributed to the volatilization of residual solvents and the decomposition of low-molecular-weight species such as unreacted imidazole. The second stage, between 179.2 and 334.4 °C, accounted for an additional 40.60% mass loss, attributed to the initial degradation of methacrylic groups, chain scission, and early decomposition of the EGDMA network.^41,44,45^ The third stage, observed between 356.1 and 458.1 °C, resulted in a further 39.35% mass loss and is associated with the decomposition of ester groups and secondary degradation products of methacrylic acid.^41,44,45^ The final residue was 2.37%, in agreement with typical behaviors of methacrylate-based systems.

The results indicate that both CDMM and EGDMA effectively promoted the formation of well-cross-linked polymeric networks, which contributed to the good thermal stability observed in both systems. The presence of covalent cross-links between polymer chains restricts segmental mobility and increases the energy required for thermal degradation, thus enhancing heat resistance even in networks with distinct structural architectures.^46^

Since polymeric implants containing dexamethasone are already widely studied and used, the dosage employed^47^ in this work is considered safe. Therefore, a cell viability assay was conducted to evaluate the biocompatibility of the formulations in the analyzed cell lines.

The resin specimens loaded with the drug were evaluated for cell viability in the VERO cell line (ATCC CCL-81) using the MTT assay (Figure 7). According to ISO 10993–5, materials are considered potentially cytotoxic when cell viability falls below 70%.

**Figure 7:**
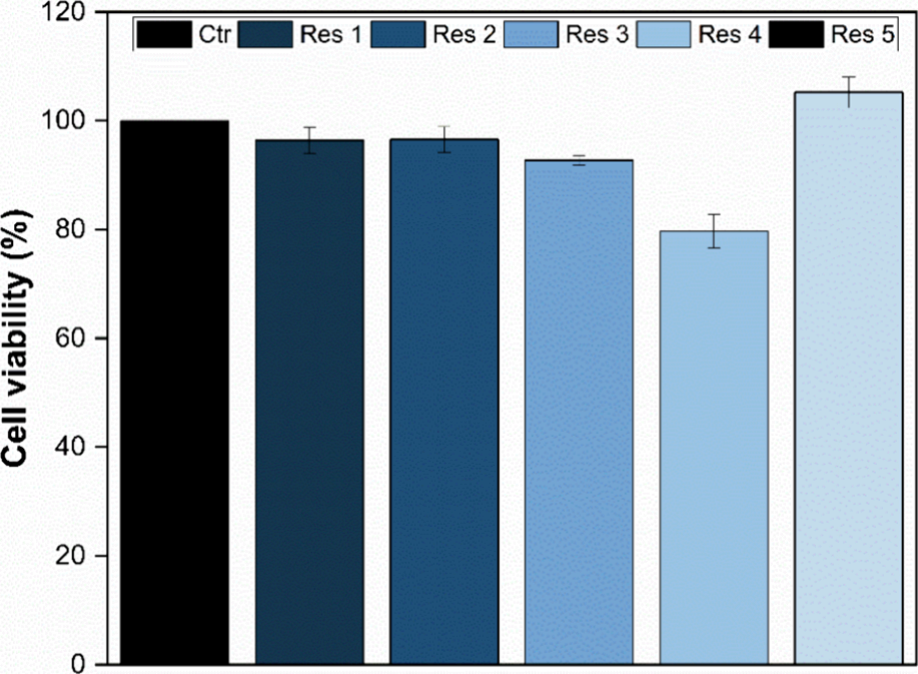
Viability (%) of the VERO cells after 24 h of exposure to resin samples loaded with dexamethasone (*n* = 3).

The results indicate that, for implants containing the CDMM cross-linker (resins 1, 2, 3, and 4), increasing the drug concentration produced only a mild effect on the cytotoxic potential of some samples, possibly due to particle agglomeration along the length of the implants. Implants 1 and 2, containing 0.126 and 0.256 mg of the drug, respectively, exhibited nearly identical cell viability (≈96.5%). Implant 3, containing 0.315 mg of dexamethasone, demonstrated a viability of 92.7%, whereas implants containing 0.630 mg of the drug showed a viability of 79.7%.

The implant formulated with EGDMA as the cross-linker and containing 0.126 mg of dexamethasone (resin 5) exhibited the highest cell viability (≈105.2%), a value commonly attributed to increased metabolic activity.^47^

Overall, all implants demonstrated cell viability above the minimum threshold established by the standard, indicating the absence of cytotoxic effects in the evaluated formulations.

The drug-loaded resin samples were also evaluated for their cytotoxic potential in corneal SIRC cells. The results, presented in Figure 8, show that implant 1, containing 0.126 mg of the drug, exhibited a cell viability of 111.2%; implant 2, containing 0.256 mg, showed 131.6%; implant 3, containing 0.315 mg, showed 105.9%; and implant 4, containing 0.630 mg of dexamethasone, exhibited 98.2%. The implant containing EGDMA as the cross-linker and 0.126 mg of the drug showed a cell viability of 115.2%.

**Figure 8:**
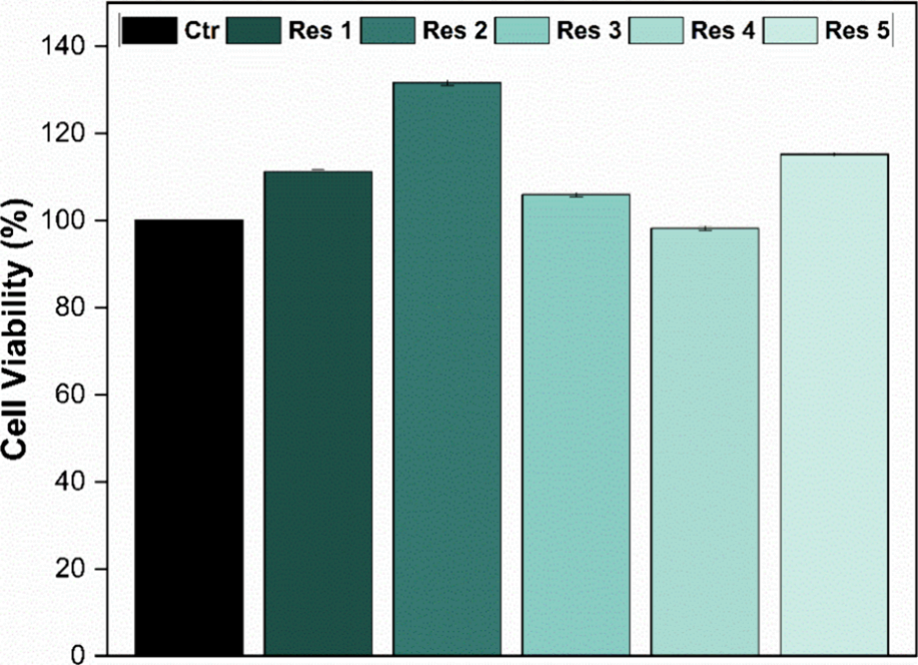
Viability (%) of corneal cells after 24 h of exposure to resin samples loaded with dexamethasone (*n* = 3).

It was observed that the initial increase in drug concentration promoted higher cell viability. However, at higher concentrations, a slight increase in cytotoxic potential was detected, possibly associated with particle agglomeration along the implant, as previously reported.^47^ This effect, however, proved to be biologically irrelevant, as all cell viability values remained above the minimum threshold established by the standard and, in most cases, exceeded those of the control group.

In other words, the implants not only exhibited no deleterious effects on the cells but also enhanced their metabolic activity, indicating excellent biocompatibility and strong potential for biomedical applications.

As a photoinitiator in our systems, 2,4,6-trimethylbenzoyl-diphenylphosphine oxide (TPO) was used at 1 mol % in the resin formulation. TPO is widely employed as a photoinitiator due to its high efficiency under near-UV and violet light, particularly around 405 nm, a wavelength commonly used in vat photopolymerization processes.^48^ In general, TPO exhibits moderate toxicity in its free form and is classified as an irritant to the skin and eyes and as potentially harmful if ingested or inhaled in significant amount. Studies indicate that TPO may exhibit concentration-dependent cytotoxicity.^48,49^ This cytotoxicity is often associated with the generation of reactive species during the photolysis process and with the interaction of aromatic fragments derived from TPO cleavage with cellular components.^48^ However, in photo-cross-linked polymeric systems, the availability of free TPO is significantly reduced after polymerization. During irradiation, TPO undergoes homolytic cleavage, generating benzoyl and phosphinoyl radicals that initiate the polymerization of double bonds. Thus, part of the initiator is consumed during initiation, while the residual fraction remains physically trapped within the formed polymer network. This entrapment, together with the high cross-linking density present in many methacrylate resins, tends to limit the diffusion and leaching of the remaining initiator.^50^

In the present study, the low TPO concentration used (approximately 0.6 mg per implant before impression) and the cross-linking density of the methacrylate-based matrices likely limit the diffusion of residual initiator species. In addition, after printing, the pieces are thoroughly washed with isopropyl alcohol and subjected to a postcuring process, which helps promote the leaching of unreacted TPO and the further reaction of TPO with remaining unpolymerized monomers.

Accordingly, the high cell viability observed in VERO cells and corneal cells indicates that any residual amounts of TPO or its photoproducts are below cytotoxic levels under the experimental conditions employed. These results suggest that TPO at this concentration is compatible with the intended application, although additional long-term biocompatibility studies will be necessary for potential clinical translation.

Once the resin was proven to be biocompatible, swelling tests and dexamethasone release studies were conducted. Figures 9 and 10 present the swelling behavior of the implants formulated with the cross-linking agents EGDMA and CDMM, both loaded with dexamethasone, as well as the drug-free formulations. The specimens were immersed in phosphate-buffered saline (PBS) for 5 days at 35.7 °C and were also evaluated at room temperature (25 °C) for comparison.

**Figure 9:**
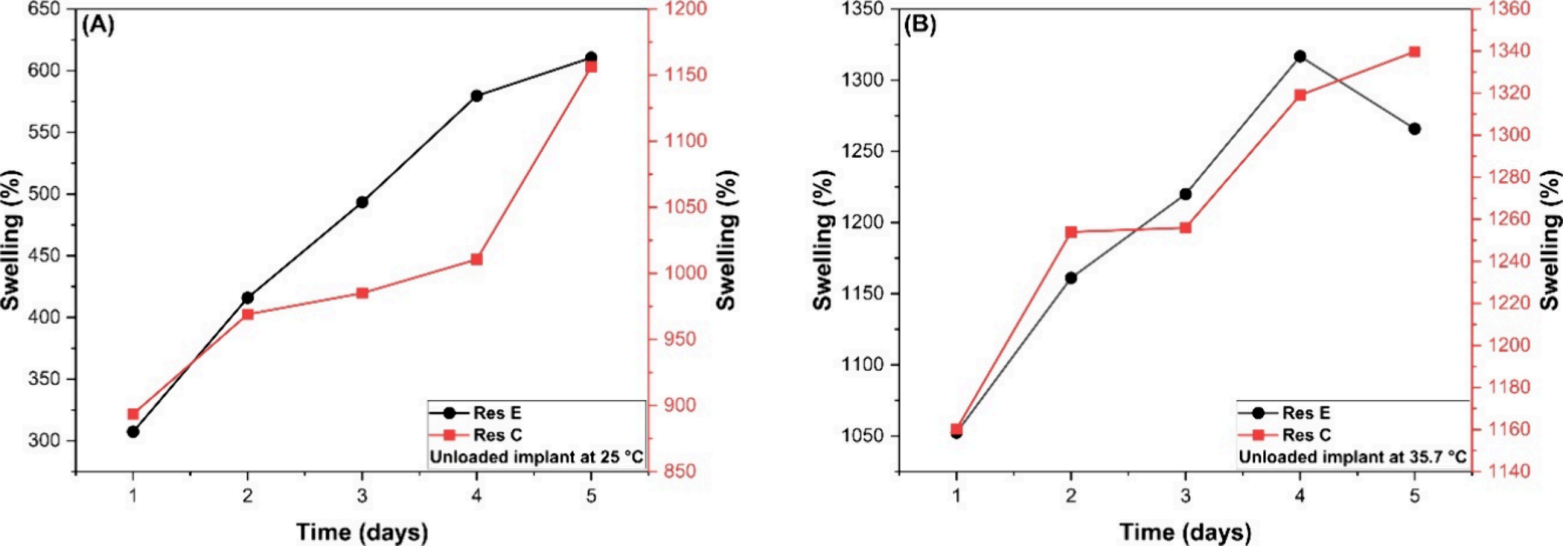
Swelling profiles of drug-unloaded implants: resin E (black) and resin C (red) at 25 °C (A) and 35.7 °C (B). The error bars are small and therefore not visible in the figures; the replicate errors are compiled in the Supporting Information.

**Figure 10:**
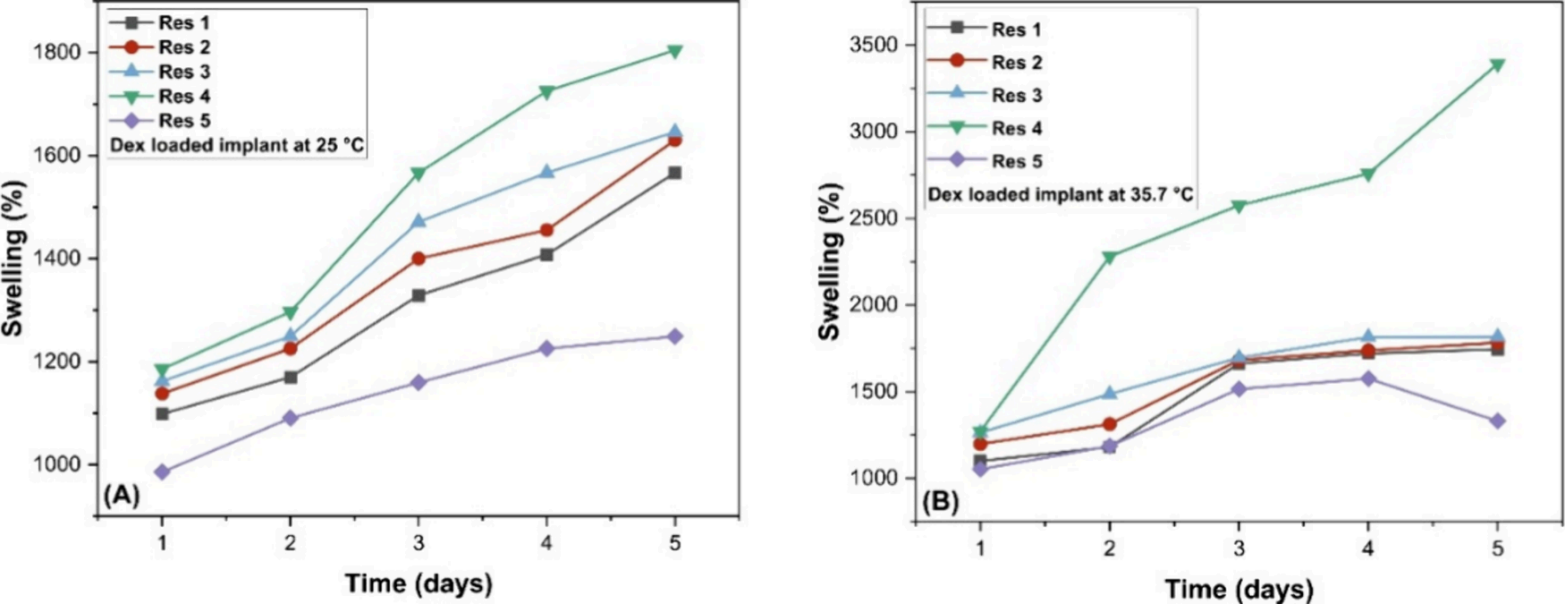
Swelling behavior of dexamethasone-loaded implants, with black representing resin 1, red resin 2, blue resin 3, green resin 4, and purple resin 5: (A) at 25 °C; (B) at 35.7 °C. The error bars are small and therefore not visible in the figures; the replicate errors are compiled in the Supporting Information.

The degree of swelling is a critical parameter in controlled drug release, particularly for ocular implants, where excessive expansion may lead to tissue damage. Swelling and deswelling behavior are characteristic of hydrogels and other biomaterials.^51,52^ During the analysis period, the implants exhibited considerable swelling, with percentage values varying according to each formulation. It was observed that increasing the drug content in the CDMM-based formulations resulted in a higher degree of swelling, indicating that the incorporation of dexamethasone directly affects the hydrophilic characteristics of the material.

The implants are composed of imidazole polymethacrylate, a cross-linking agent, and the drug. Variations in these components alter the degree of cross-linking between monomers, resulting in distinct swelling behaviors. When comparing the implants formulated with EGDMA and CDMM, both containing 40 mg of dexamethasone (res 1 and res 5, respectively; Figure 10), it was found that EGDMA promotes a higher cross-linking density among monomers, resulting in lower swelling. In contrast, CDMM exhibits greater hydrophilicity and a less densely cross-linked network, which enhances water absorption and swelling.

For a more detailed analysis, the implants were evaluated individually at the two test temperatures (25 and 35.7 °C). In these assays, the EGDMA-based implant without the drug (res E) exhibited a volume increase of approximately 610% at room temperature and 1265% when heated to 35.7 °C. The implant containing dexamethasone (res 5) showed a more pronounced swelling, with increases of 1249 and 1330%, respectively.

For the CDMM-based implants loaded with dexamethasone (res 1, res 2, res 3, and res 4), the swelling values at room temperature ranged from 1566 to 1804%, corresponding to a progressive increase in drug concentration. When heated to 35.7 °C, these values increased to 1743, 1782, 1817, and 3392%, respectively. In the absence of the drug, the swelling values were 1156% at 25 °C and 1339% at 35.7 °C.

Overall, the results demonstrated that increasing the medium temperature led to greater implant swelling, with the most pronounced effect observed in the formulation with the highest concentration. These findings indicate that the material behaves as a temperature-sensitive polymer network.

According to the literature, the thermal behavior of thermosensitive polymers is attributed to the molecular interactions between the polymer network and the surrounding aqueous medium.^46^ As the temperature increases, the molecular mobility of both water and the polymeric network rises, which tends to weaken the hydrogen bonds between water molecules and the polymer’s polar side chains, as thermal energy promotes their disruption. However, this simultaneous effect of enhanced diffusivity and relaxation of the polymer matrix may facilitate faster water penetration into the network. Thus, although each hydrogen bond becomes less stable, the overall water uptake and swelling of the polymeric network may increase due to enhanced solvent transport and absorption within the structure.^53,54^

In addition to the swelling tests performed at pH 7.4, experiments were also carried out in PBS buffer at pH 8.0. At 25 °C, resin E, containing EGDMA as the cross-linker, exhibited 617% swelling in the absence of the drug, whereas resin 5 (EGDMA and containing the drug) showed 1257% swelling after 5 days of experiment. At 35.7 °C, the swelling percentages were 1331 and 1336%, respectively. For the resins containing CDMM as the cross-linker, the swelling percentages at 25 and 35.7 °C were 1164 and 1346%, respectively. When loaded with dexamethasone, these values for the four resins were 1746% (resin 1), 1786% (resin 2), 1826% (resin 3), and 3398% (resin 4) at 35.7 °C.

Thus, with the exception of resin E, which showed lower swelling at 25 °C, the remaining values are quite similar at the higher pH, indicating that this change does not significantly affect the swelling behavior. The graphs and table corresponding to swelling at pH 8.0 at 25 and 37.5 °C are provided in the Supporting Information.

The application of matrix systems based on swellable polymers represents a highly promising strategy for developing modified-release formulations. This approach offers several advantages, including the ability to incorporate high drug loads, reduced manufacturing costs, the use of conventional processing equipment, and the achievement of consistent and reproducible drug release profiles.

In matrix systems, polymer hydration forms a gel that acts as a diffusion barrier, regulating the ingress of liquid into the matrix and, consequently, drug release through diffusion.^55^ Although many physical properties of a polymer are determined by its original structural characteristics, modifying its side chains through chemical reactions, such as cross-linking, allows these properties to be tailored to meet controlled release requirements. The structures formed by cross-linking swellable polymers are characterized by their ability to swell in water or biological fluids.^56^ The permeability of these polymers to water, drugs, and other solutes can be adjusted by modifying synthesis conditions or through polymer modification processes. This flexibility serves as a valuable tool for modulating the drug release properties.

After producing the implants with swelling capability and suitable physicochemical properties, the drug release profile was evaluated for each of the developed implants. This assay aimed to investigate the influence of the structural and compositional characteristics of the matrices on the release kinetics, allowing the correlation of the swelling behavior and material properties with the efficiency of the controlled release system.

For the dexamethasone release tests, the stock solution (0.4 mg/mL) was diluted with PBS buffer (pH 7.4) to prepare a series of standard dexamethasone solution concentrations ranging from 0.00024 to 0.015 mg/mL. Absorbances were measured, and the calibration curve was constructed, with the regression equation determined. As shown in the curve in Figure 11, the linear equation was: *y* = 164.20*x* + 0.0082, and the correlation coefficient (*R* ^2^) was 0.9990, indicating excellent linearity.

**Figure 11:**
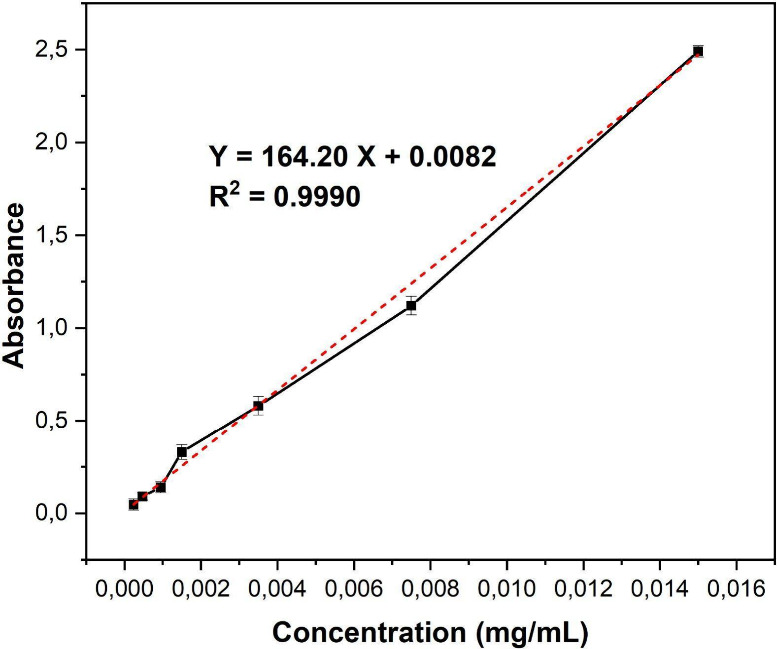
Dexamethasone calibration curve (mean ± standard deviation, *n* = 3).

To deepen the understanding of controlled drug release, the release profiles of implants containing different dexamethasone concentrations were periodically monitored until complete release was achieved. As shown in Figure 12, these profiles are influenced by both the amount of incorporated dexamethasone and the structure of the cross-linking agent. Higher drug concentrations, as well as structural differences in the cross-linker, result in faster dexamethasone release.

**Figure 12:**
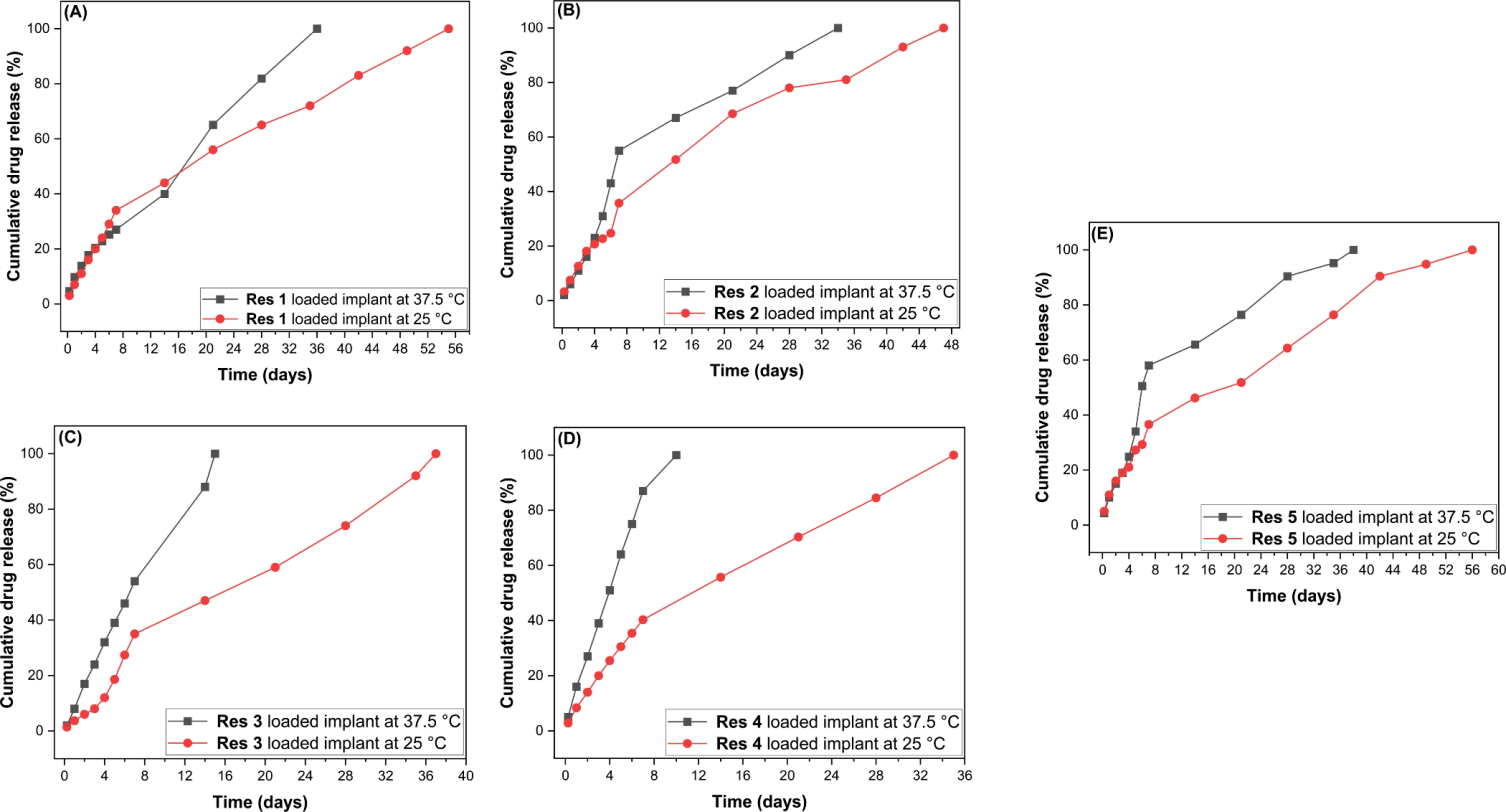
Dexamethasone release profiles from polymeric implants: (A) CDMM with 0.126 mg; (B) CDMM with 0.252 mg; (C) CDMM with 0.315 mg; (D) CDMM with 0.690 mg; and (E) EGDMA with 0.126 mg of dexamethasone per implant (mean ± standard deviation, *n* = 3). The error bars are small and therefore not visible in the figures; the replicate errors are compiled in the Supporting Information.

Drug release studies were conducted at 25 and 35.7 °C, and all developed implants exhibited complete drug release within the evaluated period. It was observed that, both at room temperature and physiological temperature, increasing the amount of dexamethasone in the implants accelerated the release process, resulting in faster kinetics.

As expected, the implants containing EGDMA as the cross-linking agent displayed more sustained release compared to those formulated with CDMM and containing 40 mg of the drug. This behavior is consistent with the previously discussed results: CDMM-cross-linked systems exhibited greater swelling and a more porous morphology than those formulated with EGDMA. These characteristics directly influence the release rate.

The times required for complete release varied according to drug concentration and temperature. For each implant containing CDMM, the results were as follows:

- **CDMM + 0.126 mg:** 55 days at 25 °C and 36 days at 35.7 °C;
- **CDMM + 0.252 mg:** 47 days at 25 °C and 35 days at 35.7 °C;
- **CDMM + 0.315 mg:** 37 days at 25 °C and 15 days at 35.7 °C;
- **CDMM + 0.690 mg:** 35 days at 25 °C and 10 days at 35.7 °C.

For each implant containing EGDMA, the result was:

- **EGDMA + 0.126 mg:** 57 days at 25 °C and 38 days at 35.7 °C.

These amounts were estimated based on the amount of drug per mL of resin and the volume of each printed piece. Since each piece has a volume of 0.0126 mL, the amount of drug per piece varies with the dexamethasone concentration per mL of resin. Because the pieces are printed using light at 405 nm and the printing medium contains acrylates together with a radical initiator, dexamethasone could, in principle, undergo other reactions, such as photodegradation or addition to the double bond. To investigate whether dexamethasone remained intact at the end of the printing process, a sample destruction assay was designed. In this test, a 2.5 × 2.5 mm specimen was macerated until complete pulverization, then immersed in PBS buffer and subjected to ultrasonic treatment for 30 min to ensure complete extraction of dexamethasone. After spectrophotometric analysis using the previously obtained dexamethasone calibration curve in PBS, it was determined that the printed specimens retained at least 95% of the drug after sample destruction. This result indicates that dexamethasone remains largely intact and available for release, based on the estimated implant volume (Table 4).

**Table 4:** Recovery Rates from Immediate Release Tests

**resin designation**	**estimated amount of drug in each implant** (in mg, 100%)	**drug recovered (%)**	**mass of recovered drug (mg)**
Res 1	0.126	95.3 ± 0.8	0.121 ± 0.001
Res 2	0.252	98.1 ± 0.4	0.247 ± 0.001
Res 3	0.315	98.3 ± 0.7	0.309 ± 0.002
Res 4	0.690	96.8 ± 0.4	0.668 ± 0.002
Res 5	0.126	97.6 ± 0.8	0.123 ± 0.001

Overall, increasing the drug concentration and the use of CDMM favored faster release, particularly under physiological conditions. A higher drug concentration in controlled-release devices may enhance the release rate due to the formation of more pronounced concentration gradients that promote drug diffusion. This principle is particularly relevant in diffusion-based controlled-release systems, such as polymeric matrices and reservoir structures. Studies on release kinetics indicate that variations in the initial drug concentration have a significant impact on the release profile over time.^57^

In recent years, several studies have investigated how the initial drug concentration in controlled devices influences the release rate. For example, Rashid et al.^58^ demonstrated in studies using polymeric hydrogels such as PHEMA (polyhydroxyethyl methacrylate) that increase the drug concentration, intensifying its release by altering solute transport kinetics. This behavior has been observed in studies on drug–polymer interactions and controlled release in biomedical applications, including implants.^59^

Devices based on temperature- or pH-responsive acrylate systems, such as those composed of PNIPAAm, poly­(*N*-isopropylacrylamide), also demonstrate that drug concentration influences release rates. The behavior of these polymers, combined with factors such as temperature and pH, is adjusted to optimize the release profile, particularly in clinically sensitive environments.^60^ These studies highlight how the design of controlled-release systems can be modulated to meet therapeutic demands by manipulating or increasing the initial drug concentration.

Regarding temperature, recent research indicates that increased temperature can accelerate drug release in controlled systems, especially those based on biodegradable polymers. This occurs because heat intensifies processes such as diffusion, drug solubility, and degradation of the device material, all of which are critical to release. For example, nanoparticulate systems exhibit significantly higher release rates when exposed to elevated temperatures due to increased matrix dissolution and drug permeability.^61^

Moritz et al.^62^ emphasize that polymeric controlled-release systems can be engineered to respond to stimuli such as temperature. Increasing temperature can modify polymeric properties such as hydration and swelling, thereby accelerating drug release in heated environments.

The application of knowledge regarding the influence of concentration and temperature on drug release is essential for the development of ocular drug-delivery devices, such as therapeutic lenses or controlled-release implants. These devices must be designed considering the specific conditions of the human eye, a complex and dynamic environment. Adjusting the drug concentration in the device enables optimization of the diffusion gradient, which is crucial for determining the release rate and profile.

In ocular systems, this is particularly important due to the limited retention capacity of the ocular surface and the rapid drainage of tear fluid. An optimal initial concentration ensures a sustained and effective release, minimizing losses and maximizing the therapeutic effect. Ocular temperature may vary, especially in cases of inflammation or infection, and temperature-responsive devices can adjust release accordingly. This feature is important for treating conditions in which elevated temperature may indicate an increased need for medication.^46^ The combined understanding of concentration and temperature effects in the formulation of ocular devices enhances therapeutic precision and reduces the risk of adverse effects. This approach not only improves the treatment of ocular diseases but also favors greater patient comfort and adherence.^46^ Thus, strategies based on concentration and temperature emerge as a fundamental innovation in the design of ocular controlled-release systems.

The drug release data were analyzed according to the diffusion model proposed by Korsmeyer–Peppas (Table 5). All systems exhibited high determination coefficients (*R* ^2^ = 0.95–0.99), indicating an excellent fit of the model to the experimental release profiles at both 25 and 35.7 °C.

**Table 5:** Korsmeyer–Peppas Kinetic Model Parameters

	**Korsmeyer–Peppas kinetic model**					
	at 25 °C			at 35.7 °C		
**resins**	***R*** ^**2**^	***n***	***k***	***R*** ^**2**^	***n***	***k***
Res 1	0.96	0.73	0.0830	0.99	0.78	0.0655
Res 2	0.97	0.82	0.0284	0.95	0.81	0.0645
Res 3	0.97	0.65	0.0837	0.99	0.99	0.0632
Res 4	0.97	0.98	0.0433	0.98	0.98	0.127
Res 5	0.97	0.68	0.0845	0.95	0.59	0.109

At 25 °C, the release exponent *n* ranged from 0.65 to 0.98, suggesting mechanisms that span from anomalous transport (a combination of diffusion and polymer relaxation) to behavior close to Case II transport, particularly for Resin 4 (*n* = 0.98). At 35.7 °C, a general increase in *n* values was observed, indicating enhanced polymer relaxation processes under physiological conditions. At this temperature, Resins 3 and 4 stood out with high *n* values (*n* = 0.99 and 0.98, respectively), characterizing a release mechanism predominantly governed by matrix relaxation and swelling.

In the Korsmeyer–Peppas model, the constant *k* is characteristic of each delivery system and depends on the polymer type, temperature, device geometry, and other experimental conditions. In other words, *k* is an integrated structural, geometric, and kinetic parameter that defines the initial release rate; thus, the higher its value, the faster the initial release.^63^ At 25 °C, all *k* values were less than 0.1, with the highest values observed for resins 1, 3, and 5. At 35.7 °C, a temperature more relevant to this study, the highest values were associated with resins 4 and 5, corresponding to the resin containing the highest amount of drug and using CDMM as the cross-linker, and the resin containing EGDMA as the cross-linker, respectively. Particularly in the case of resin 4, which presented the highest calculated *k* value, it is worth noting that this resin also exhibited the fastest drug release and contains the highest drug loading, which is consistent with a greater initial release. These results confirm that the release mechanism is sensitive to both the cross-linker used in the polymeric matrix and the temperature, highlighting the combined influence of polymer structure, cross-linking agent, and chain mobility on the release kinetics.

The *k* values obtained here were relatively low compared to some reports in the literature. For example, PVA hydrogels containing dexamethasone at different concentrations have exhibited *k* values above 1.0, reaching values higher than.^64^ Szalai and co-workers, who also developed systems for ocular implants, reported *k* values above 1.0, reaching up to 25.0, depending on the delivery system (gel or polymeric micelle).^65^ These findings indicate that our systems provide a more sustained release, since the *k* values calculated here are significantly lower, suggesting the absence of a large initial drug burst.

Although other diffusion models could also be explored,^66,67^ the Korsmeyer–Peppas model showed a good correlation with the release rates and provided a mechanism consistent with that reported in other studies involving dexamethasone incorporated into polymeric matrices. The Higuchi model, for example, showed an *R* ^2^ value of 0.93 for resin 2, indicating that the Korsmeyer–Peppas model provides a better fit to the experimental data. Other models, such as the Hixson-Crowell model, were not applied because they are not considered suitable for the conditions of our experiments, in which there is no loss of polymer mass during release nor a reduction in particle surface area;^68^ instead, the process is governed by matrix relaxation.

Nevertheless, other studies in which dexamethasone was used as a model drug in polymeric delivery systems reported Fickian or pseudo-Fickian diffusion,^69,70^ with *n* values below 0.5, indicating diffusion-controlled release rather than matrix relaxation, as observed in our work.

## Conclusions

Networks based on imidazolium methacrylate were obtained and cross-linked with either cyclohexanedimethanol dimethacrylate (CDMM) or ethylene glycol dimethacrylate (EGDMA), exhibiting suitable mechanical properties, satisfactory thermal stability, and in vitro biocompatibility in VERO cells and corneal cells. The results demonstrated that the release of dexamethasone from the device is primarily governed by a combined mechanism of diffusion and relaxation of the polymeric matrix, with the predominance varying according to the network composition and the conditions of the surrounding medium. It was shown that the nature of the cross-linker exerts a decisive influence on the structural organization of the matrix and, consequently, on the release profiles: agents with lower steric hindrance favored higher cross-linking density and reduced release rates, whereas higher drug loadings increased porosity and accelerated transport kinetics. Increasing temperature enhanced swelling and chain mobility, confirming the system’s sensitivity to external stimuli. In this study, five implant models with distinct profiles for dexamethasone-controlled release were developed.

Taken together, the findings demonstrate that the structural modulation of photopolymerizable networks allows the rate and mechanism of dexamethasone release to be predictably adjusted by manipulating variables such as cross-linker type, cross-linking density, drug loading, and temperature. All developed systems exhibited high cell viability, indicating preliminary suitability for intraocular biomedical applications.

Therefore, the proposed polymeric matrices can be regarded as promising platforms for sustained intravitreal dexamethasone release, with the potential to reduce the frequency of repeated administrations and minimize limitations associated with prolonged systemic therapy.

## Supplementary Material



## Notes

^1^H, ^13^C NMR, UV–vis, and FTIR spectroscopy and mass spectrometry data; photograph of the printer adapted with mini vat and mini platform; and tensile tests, dissolution profiles, average standard deviation of cumulative drug release and for all swelling data, and swelling data at pH 8 (PDF)
