# Supplementary material for: Photopolymerizable Imidazolium Methacrylate Networks for Controlled Dexamethasone Release: Potential Application in Uveitis Therapy
Source: ACS Omega. 2026 Apr 27;11(18):26553–68. doi: 10.1021/acsomega.5c12938 (PMC13176969; doi:10.1021/acsomega.5c12938)
Supplement: Supplementary file 1 [file ao5c12938_si_001.pdf]

SUPPORTING INFORMATION

Photopolymerizable Imidazolium Methacrylate Networks for Controlled Dexamethasone Release: Potential Application in Uveitis Therapy

Maria J. S. Lima, Dayane K. D. N. Santos, Janaína V. dos Anjos*, Severino Alves-Jr*

Departamento de Química Fundamental, Universidade Federal de Pernambuco,

50740-560 Recife, PE, Brazil

*janaina.anjos@ufpe.br

*severino.alvesjr@ufpe.br

Table of contents:

1. ^{1}H and ^{13}C NMR spectra	S3
2. Photograph of the printer adapted with mini vat and mini platform	S5
3. UV-Vis spectra	S6
4. FTIR spectra	S6
5. Tensile Tests	S9
6. Dissolution profile	S12
7. Average standard deviation of cumulative drug release	S13
8. Average standard deviation for swelling	S13
9. Swelling percentage at pH 8	S14

Abbreviations

EGDMA – Ethylene glycol dimethacrylate

CDMM – Cyclohexanedimethanol-dimetacrylate

Res C – Implant with CDMM crosslinker and without drug

Res E – Implant with EGDMA crosslinker and without drug

Res 1 – Implant containing 0,126 mg of dexamethasone and CDMM crosslinker

Res 2 – Implant containing 0,252 mg of dexamethasone and CDMM crosslinker

Res 3 – Implant containing 0,315 mg of dexamethasone and CDMM crosslinker

Res 4 – Implant containing 0,690 220 mg of dexamethasone and CDMM crosslinker

Res 5 – Implant containing 0,126 mg of dexamethasone and EGDMA crosslinker

^{1}H and ^{13}C NMR spectra

Figure S1. ^{1}H NMR Spectrum ($CDCl_3$, 400 MHz) **EGDMA**

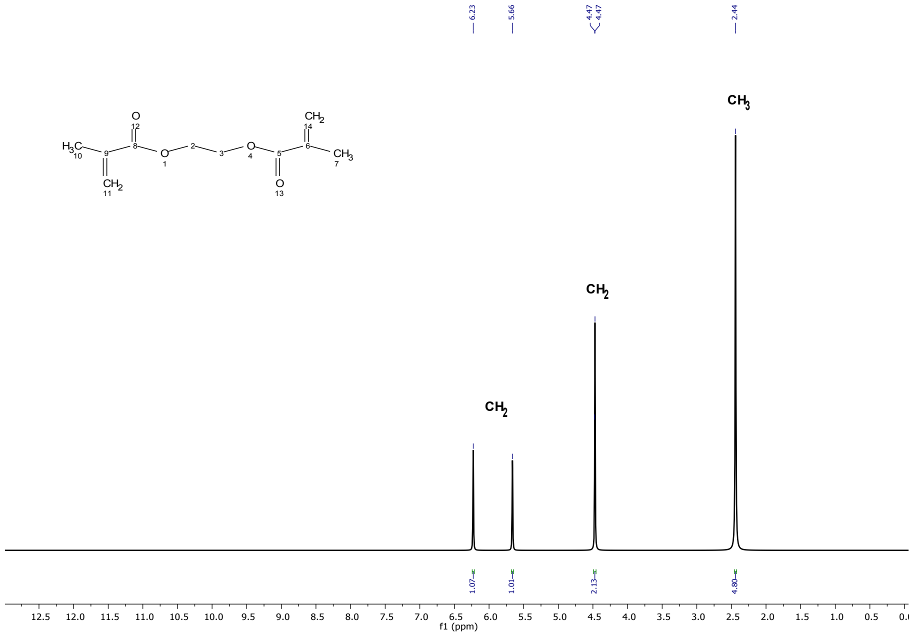

Figure S2. ^{13}C NMR Spectrum ($CDCl_3$, 100 MHz) **EGDMA**

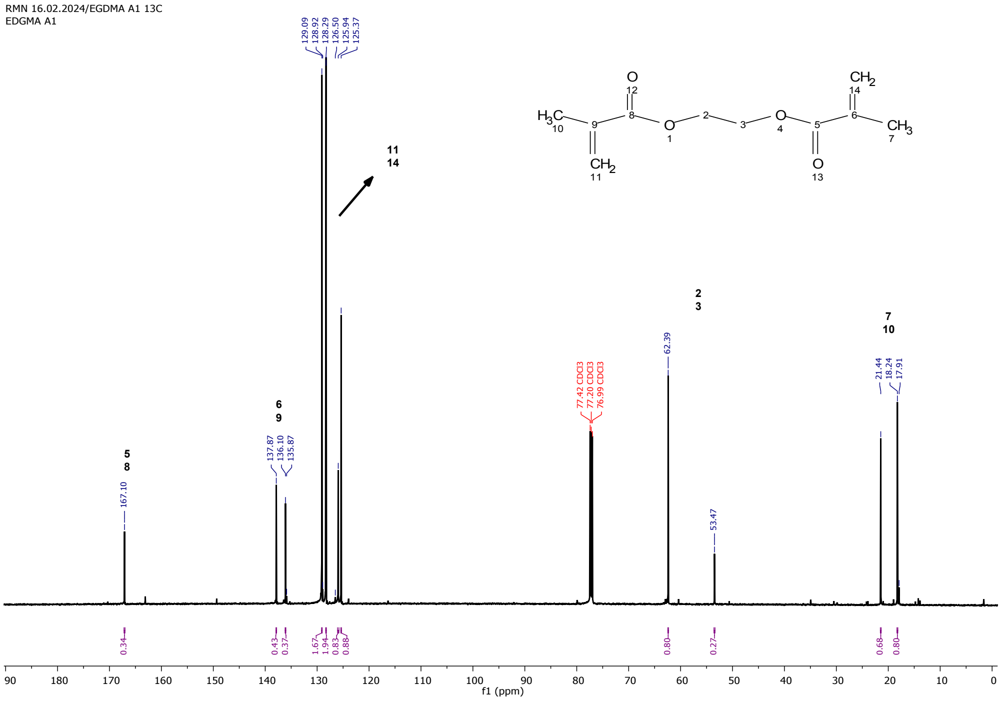

Figure S3. ^{1}H NMR Spectrum ($CDCl_3$, 400 MHz) **CDMM**

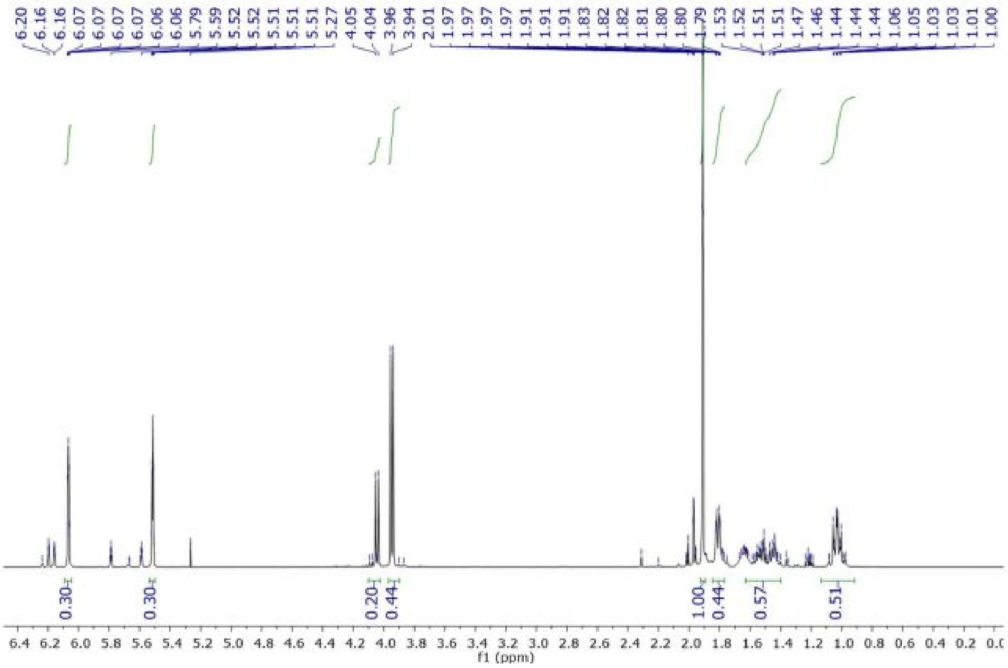

Figure S4. ^{13}C NMR Spectrum ($CDCl_3$, 100 MHz) **CDMM**

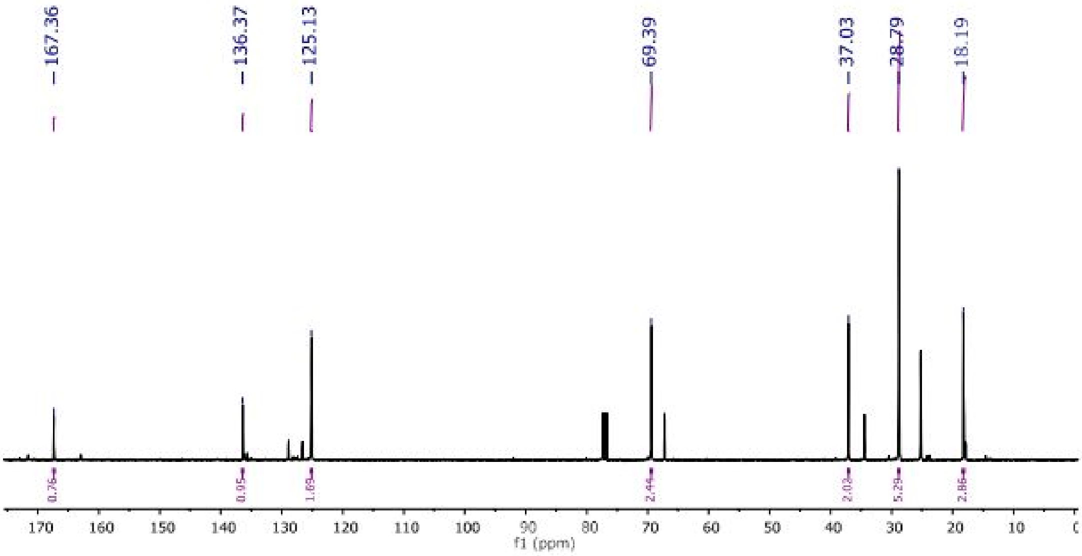

Figure S5. ^{1}H NMR Spectrum (DMSO, 400 MHz) **DEXAMETHASONE**

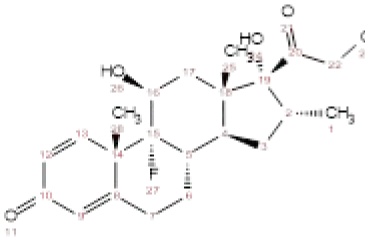

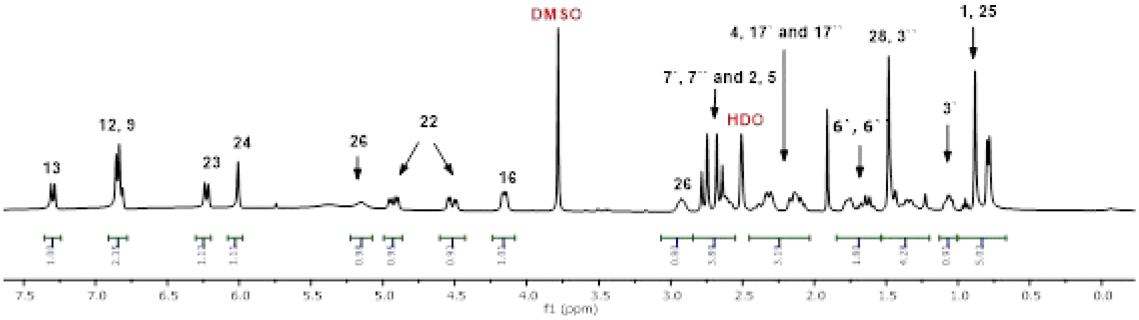

MLSA printer

Figure S6. Photograph of the printer adapted with a mini vat and a mini platform

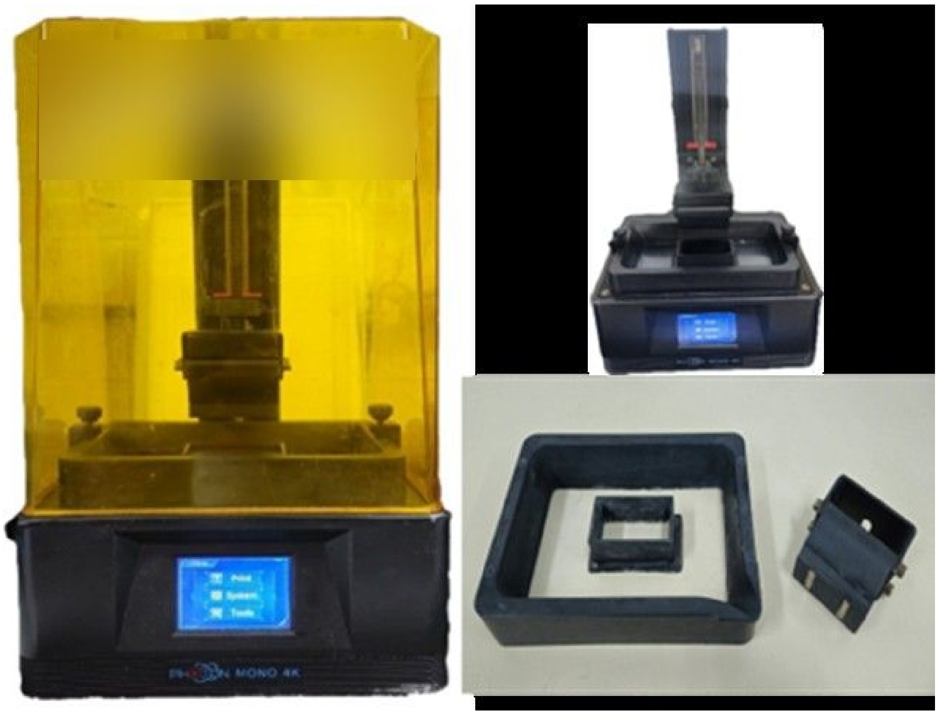

UV-Vis Spectra

Figure S7: Maximum absorption wavelength of dexamethasone: A) obtained in this study B) reported in the literature

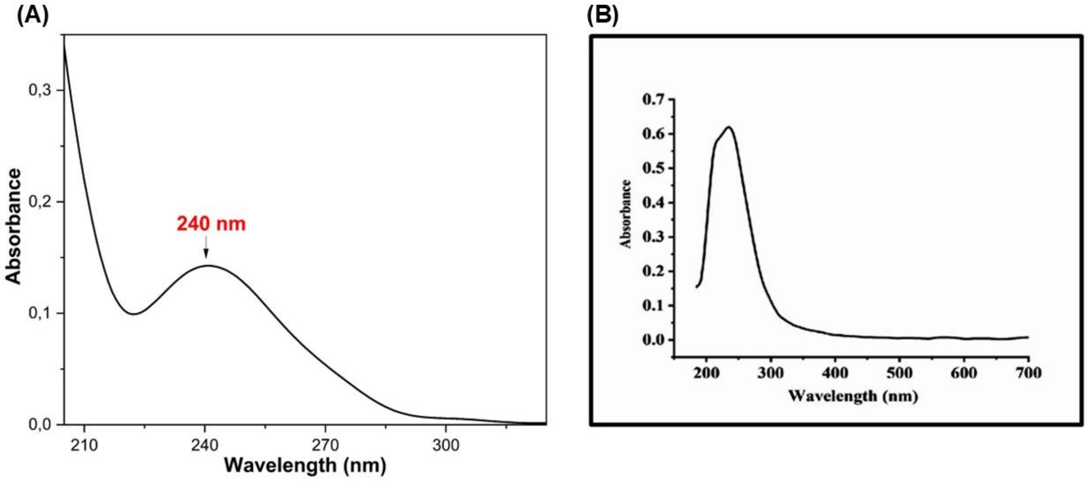

Source: DOI: 10.1007/s00296-025-06030-y

FTIR spectra

Figure S8: FTIR spectrum of methacrylic acid

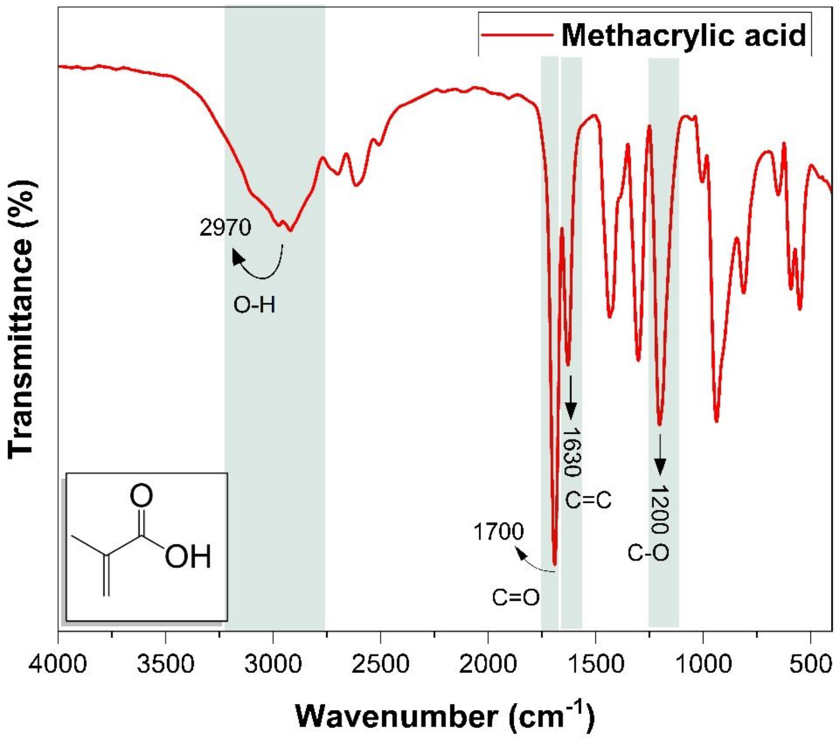

Figure S9: FTIR spectrum of EGDMA

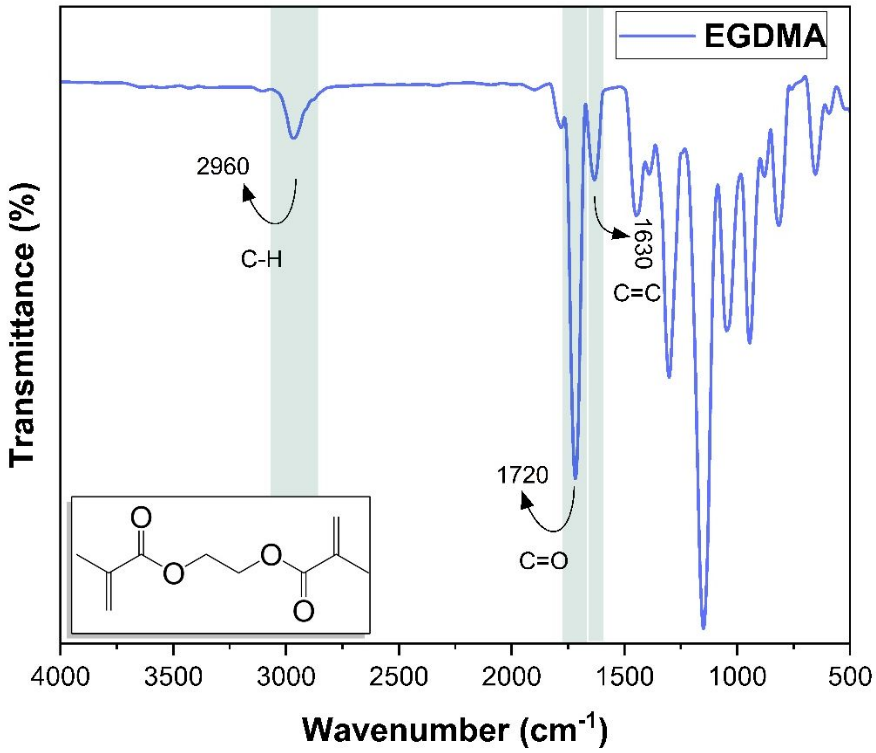

Figure S10: FTIR spectrum of CDMM

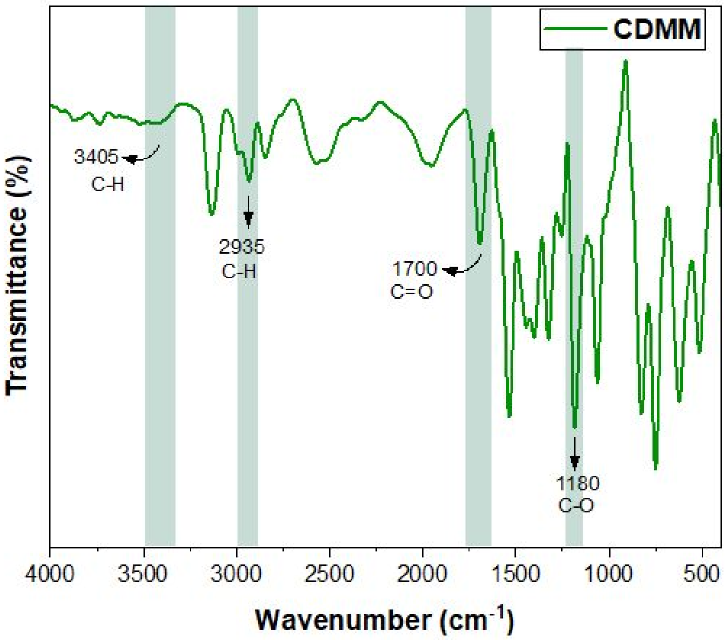

Figure S11: FTIR spectrum of Imidazole

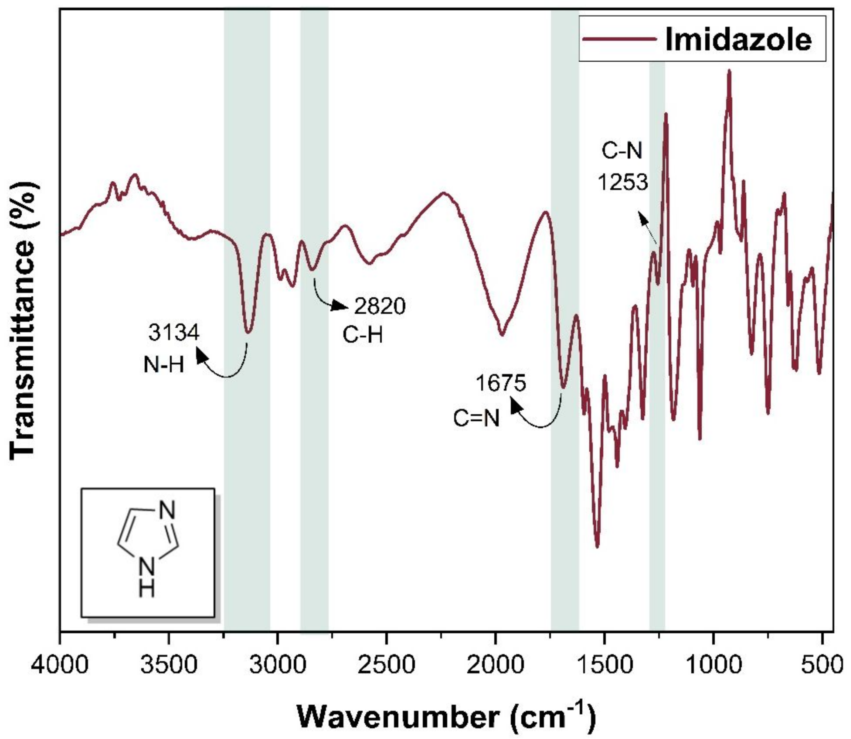

Figure S12: FTIR spectrum of dexamethasone: A) obtained in this study; B) reported in the literature

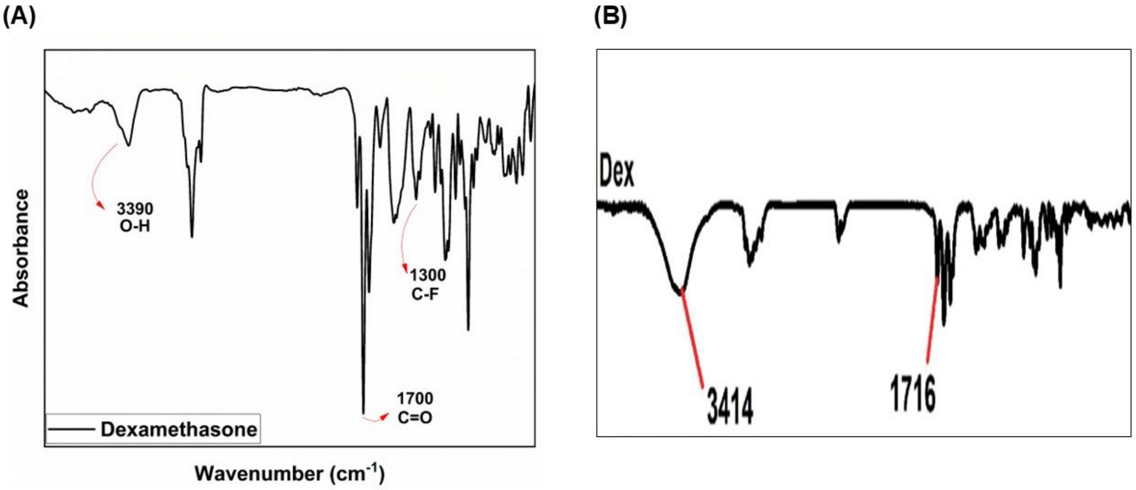

Source: DOI:10.3109/10717544.2013.801049

Tensile Tests

Figure S13: Tensile Test Report for Post-Printed Resin C

Máquina: **Emic -** Célula: **Trd 22** Extensômetro: - Data: **08/08/2024** Hora: **11:55:54** Trabalho nº **3433**

Programa: **Tesc versão 3.04** Método de Ensaio: **Ensaio de Tração**

Ident. Amostra: >>>>>>>>>>>>>>>>>>>>>>>>>>>>>>>> Corpo de Prova: **C1** Responsável : **MARIA LIMA** Operador: **Rubens**

Corpo de Prova	Largura (mm)	Espessura (mm)	Compr. Base (mm)	Tensão @Força Max. (MPa)	Tensão @Ruptura (MPa)	Mod.Elástic. (MPa)	Def.Especif. @Ruptura (%)
CP 1	1.78	1.94	12.50	24	23.53	1518.72	4.09
CP 2	1.78	1.94	12.50	25	23.80	1385.13	8.34
CP 3	*	*	*	*	*	*	*
CP 4	1.78	1.94	12.50	33	32.78	1575.31	4.40

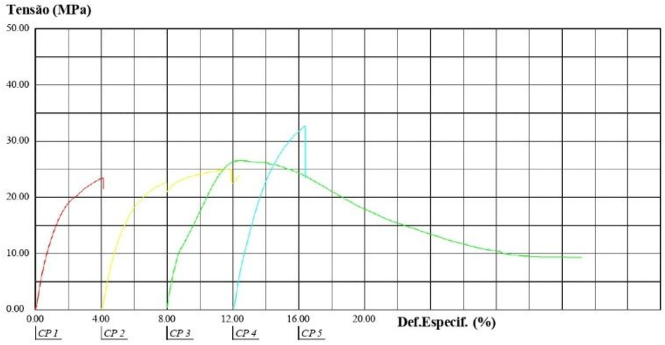

Figure S14: Tensile Test Report for Post-Printed Resin E

Máquina: **Emic -** Célula: **Trd 22** Extensômetro: - Data: **08/08/2024** Hora: **11:44:15** Trabalho nº **3432**

Programa: **Tesc versão 3.04** Método de Ensaio: **Ensaio de Tração**

Ident. Amostra: >>>>>>>>>>>>>>>>>>>>>>>>>>>>>>>> Corpo de Prova: **E1** Responsável : **MARIA LIMA** Operador: **Rubens**

Corpo de Prova	Largura (mm)	Espessura (mm)	Compr. Base (mm)	Tensão @Força Max. (MPa)	Tensão @Ruptura (MPa)	Mod.Elástic. (MPa)	Def.Especif. @Ruptura (%)
CP 1	1.78	1.94	12.50	18	18.18	1152.36	2.53
CP 2	1.78	1.94	12.50	12	11.57	637.15	2.75
CP 3	*	*	*	*	*	*	*
CP 4	1.78	1.94	12.50	11	11.08	649.49	1.56

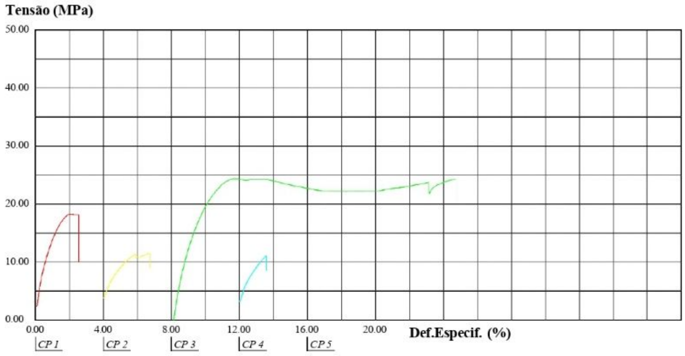

Figure S15: Tensile Test Report for Post-Printed Resin 1

Máquina: **Emic** - Célula: **Trd 22** Extensômetro: - Data: **08/08/2024** Hora: **11:27:30** Trabalho nº **3431**
Programa: **Tesc versão 3.04** Método de Ensaio: **Ensaio de Tração**
Ident. Amostra: >>>>>>>>>>>>>>>>>>>>>>>>>>>>>>>>> Corpo de Prova: **C40** Responsável: **MARIA LIMA** Operador: **Rubens**

Corpo de Prova	Largura (mm)	Espessura (mm)	Compr. Base (mm)	Tensão @Força Max. (MPa)	Tensão @Ruptura (MPa)	Mod.Elástic. (MPa)	Def.Especif. @Ruptura (%)
CP 1	**1.78**	**1.94**	**12.50**	**69**	**68.72**	**1725.31**	**8.31**
CP 2	*	*	*	*	*	*	*
CP 3	*	*	*	*	*	*	*
CP 4	**1.78**	**1.94**	**12.50**	**29**	**28.70**	**1609.12**	**2.61**
CP 5	**1.78**	**1.94**	**12.50**	**28**	**28.25**	**1744.95**	**2.42**

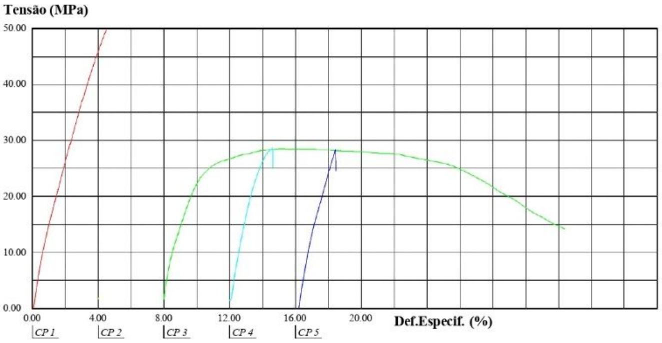

Figure S16: Tensile Test Report for Post-Printed Resin 2

Máquina: **Emic** - Célula: **Trd 22** Extensômetro: - Data: **08/08/2024** Hora: **11:14:52** Trabalho nº **3430**
Programa: **Tesc versão 3.04** Método de Ensaio: **Ensaio de Tração**
Ident. Amostra: >>>>>>>>>>>>>>>>>>>>>>>>>>>>>>>>> Corpo de Prova: **C80** Responsável: **MARIA LIMA** Operador: **Rubens**

Corpo de Prova	Largura (mm)	Espessura (mm)	Compr. Base (mm)	Tensão @Força Max. (MPa)	Tensão @Ruptura (MPa)	Mod.Elástic. (MPa)	Def.Especif. @Ruptura (%)
CP 1	**1.78**	**1.94**	**12.50**	**29**	**29.34**	**1328.40**	**3.94**
CP 2	*	*	*	*	*	*	*
CP 3	**1.78**	**1.94**	**12.50**	**39**	**39.07**	**2157.44**	**2.74**
CP 4	**1.78**	**1.94**	**12.50**	**37**	**37.03**	**1593.16**	**2.77**

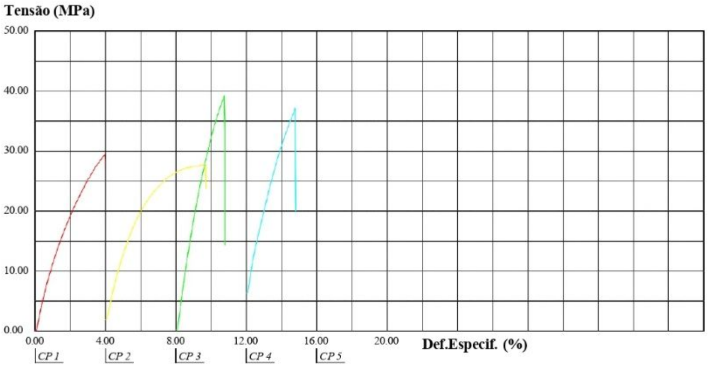

Figure S17: Tensile Test Report for Post-Printed Resin 3

Máquina: **Emic** - Célula: **Trd 22** Extensômetro: - Data: **08/08/2024** Hora: **10:13:09** Trabalho nº **3428**

Programa: **Tesc versão 3.04** Método de Ensaio: **Ensaio de Tração**

Ident. Amostra: >>>>>>>>>>>>>>>>>>>>>>>>>>>>>>>> Corpo de Prova: **C100** Responsável: **MARIA LIMA** Operador: **Rubens**

Corpo de Prova	Largura (mm)	Espessura (mm)	Compr. Base (mm)	Tensão @Força Max. (MPa)	Tensão @Ruptura (MPa)	Mod.Elástic. (MPa)	Def.Especif. @Ruptura (%)
CP 1	1.78	1.94	12.50	29	*	1582.25	*
CP 2	*	*	*	*	*	*	*
CP 3	1.78	1.94	12.50	37	*	1923.65	*
CP 4	1.78	1.94	12.50	43	*	1780.10	*
CP 5	*	*	*	*	*	*	*

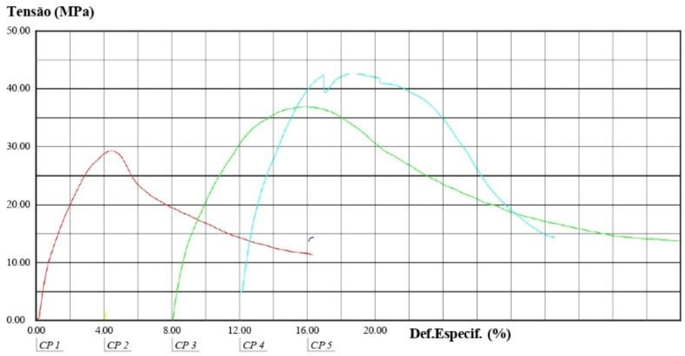

Figure S18: Tensile Test Report for Post-Printed Resin 4

Máquina: **Emic** - Célula: **Trd 22** Extensômetro: - Data: **08/08/2024** Hora: **10:45:20** Trabalho nº **3429**

Programa: **Tesc versão 3.04** Método de Ensaio: **Ensaio de Tração**

Ident. Amostra: >>>>>>>>>>>>>>>>>>>>>>>>>>>>>>>> Corpo de Prova: **C220** Responsável: **MARIA LIMA** Operador: **Rubens**

Corpo de Prova	Largura (mm)	Espessura (mm)	Compr. Base (mm)	Tensão @Força Max. (MPa)	Tensão @Ruptura (MPa)	Mod.Elástic. (MPa)	Def.Especif. @Ruptura (%)
CP 1	1.78	1.94	12.50	40	*	1848.50	*
CP 2	1.78	1.94	12.50	40	*	1835.59	*
CP 3	1.78	1.94	12.50	28	28.49	1607.91	5.83

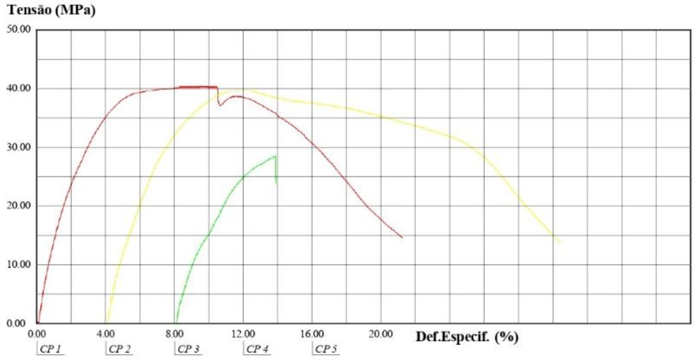

Figure S19: Tensile Test Report for Post-Printed Resin 5

Máquina: **Emic** - Célula: **Trd 22** Extensômetro: - Data: **08/08/2024** Hora: **12:19:52** Trabalho nº **3435**

Programa: **Tesc versão 3.04** Método de Ensaio: **Ensaio de Tração**

Ident. Amostra: >>>>>>>>>>>>>>>>>>>>>>>>>>>>>>>>>> Corpo de Prova: **E40** Responsável : **MARIA LIMA** Operador: **Rubens**

Corpo de Prova	Largura (mm)	Espessura (mm)	Compr. Base (mm)	Tensão @Força Max. (MPa)	Tensão @Ruptura (MPa)	Mod.Elástic. (MPa)	Def.Especif. @Ruptura (%)
CP 1	1.78	1.94	12.50	20	*	1327.73	*
CP 2	1.78	1.94	12.50	23	*	1958.08	*
CP 3	*	*	*	*	*	*	*
CP 4	*	*	*	*	*	*	*
CP 5	1.78	1.94	12.50	21	*	1676.19	*

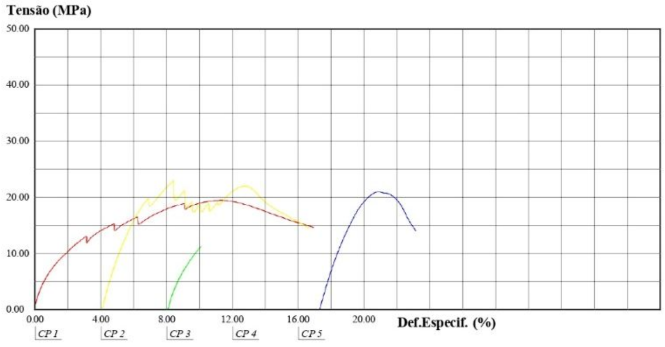

Drug dissolution profile

Figure S20: Drug dissolution profile at 35.7 °C

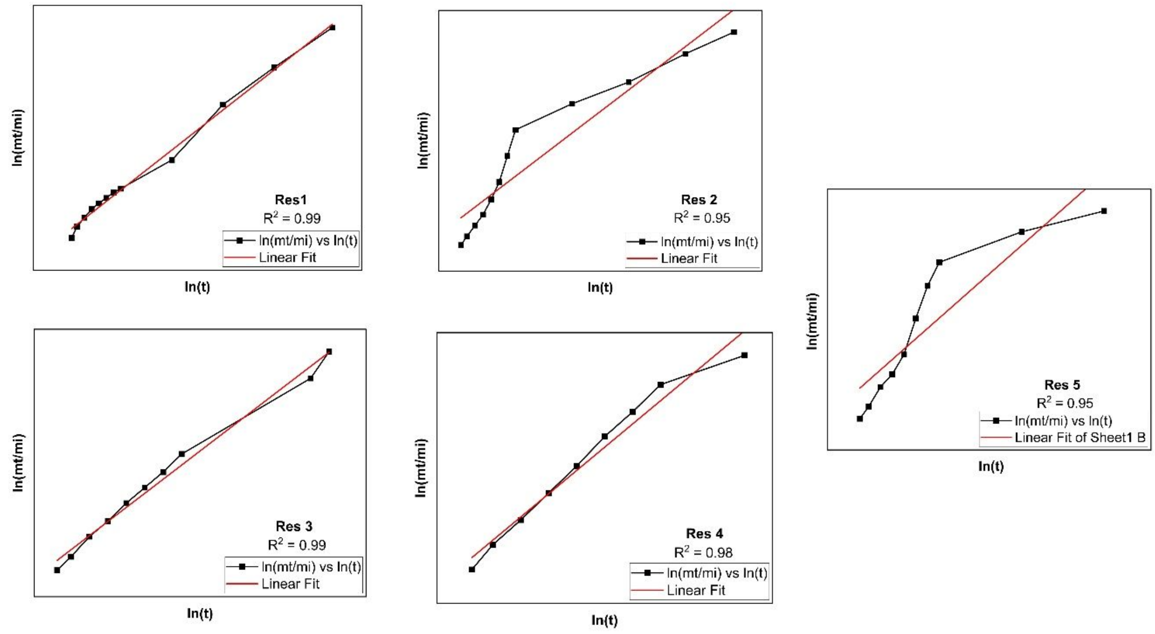

Figure S21: Drug dissolution profile at 25 °C

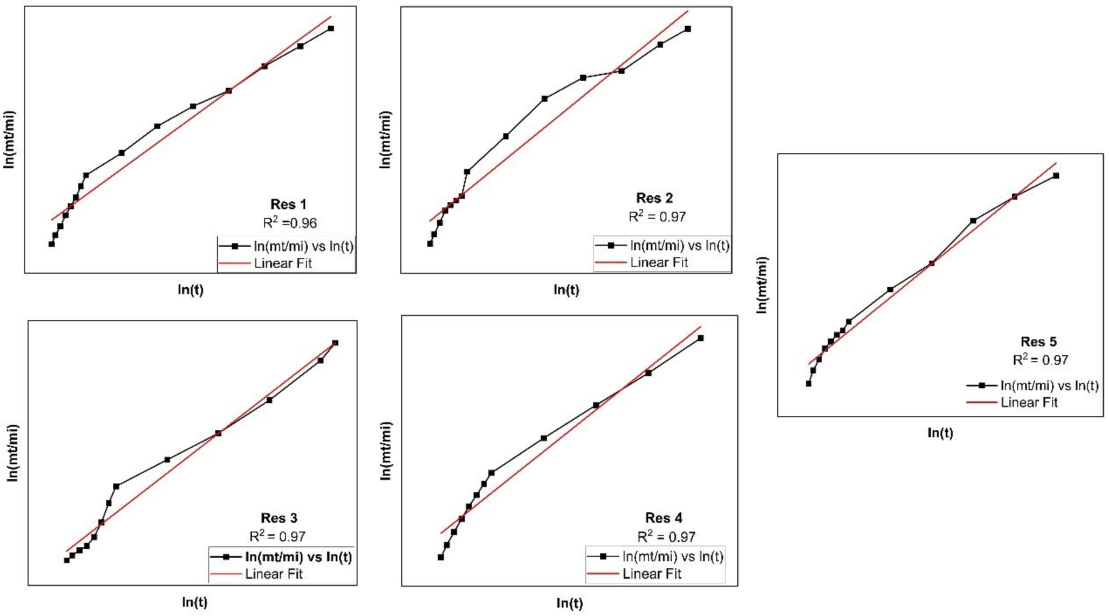

Table S1: Average standard deviation of cumulative drug release

	at 25 °C	at 35.7 °C
Res 1	± 0.00472	± 0.00312
Res 2	± 0.00258	± 0.00594
Res 3	± 0.00169	± 0.00149
Res 4	± 0.00253	± 0.00338
Res 5	± 0.00222	± 0.00159

Table S2: Average standard deviation for swelling with drug (pH 7.4)

	at 25 °C (%)	at 35.7 °C (%)
Res 1	± 5.95	± 15.5
Res 2	± 14.8	± 11.5
Res 3	± 13.1	± 8.20
Res 4	± 9.40	± 13.6
Res 5	± 12.3	± 10.4

Table S3: Average standard deviation for swelling without drug (pH 7.4)

	at 25 °C (%)	at 35.7 °C (%)
Res C	± 11.1	± 15.4
Res E	± 9.8	± 12.2

Table S4: Swelling percentage of drug-free implants at pH 8

	at 25 °C (%)		at 35.7 °C (%)	
Day	Res C	Res E	Res C	Res E
1	897	314	1170	1173
2	981	419	1254	1170
3	987	495	1260	1225
4	1012	581	1340	1326
5	1164	617	1346	1331

Table S5: Swelling percentage of drug-loaded implants at pH 8

	at 25 °C (%)						at 35.7 °C (%)				
Day	Res 1	Res 2	Res 3	Res 4	Res 5		Res 1	Res 2	Res 3	Res 4	Res 5
1	1101	1140	1165	1190	991		1105	1200	1268	1275	1055
2	1173	1235	1251	1299	1097		1184	1319	1490	2290	1190
3	1332	1404	1473	1569	1164		1669	1692	1699	2579	1517
4	1409	1459	1571	1729	1228		1730	1744	1823	2764	1582
5	1573	1638	1656	1808	1257		1746	1786	1826	3398	1336

Table S6: Average standard deviation for swelling with drug (pH 8.0)

	**at 25 °C (%)**	**at 35.7 °C (%)**
Res 1	± 13.2	± 13.3
Res 2	± 12.4	± 14.5
Res 3	± 11.2	± 13.6
Res 4	± 8.7	± 9.8
Res 5	± 9.1	± 12.1

Table S7: Average standard deviation for swelling without drug (pH 8.0)

	**at 25 °C (%)**	**at 35.7 °C (%)**
Res C	± 13.2	± 13.8
Res E	± 10.1	± 14.9
